# Opportunities, Challenges and Prospects for Electrodeposition of Thin-Film Functional Layers in Solid Oxide Fuel Cell Technology

**DOI:** 10.3390/ma14195584

**Published:** 2021-09-26

**Authors:** Elena Kalinina, Elena Pikalova

**Affiliations:** 1 Laboratory of Complex Electrophysic Investigations, Institute of Electrophysics, Ural Branch of the Russian Academy of Sciences, 620016 Yekaterinburg, Russia; 2 Department of Physical and Inorganic Chemistry, Institute of Natural Sciences and Mathematics, Ural Federal University, 620002 Yekaterinburg, Russia; 3 Laboratory of Solid Oxide Fuel Cells, Institute of High Temperature Electrochemistry, Ural Branch of the Russian Academy of Sciences, 620137 Yekaterinburg, Russia; 4 Department of Environmental Economics, Graduate School of Economics and Management, Ural Federal University, 620002 Yekaterinburg, Russia

**Keywords:** SOFC, electrodeposition, electrolytic deposition, electrophoretic deposition, thin-film technology, porous substrate, ceria, electrochemical reaction

## Abstract

Electrolytic deposition (ELD) and electrophoretic deposition (EPD) are relevant methods for creating functional layers of solid oxide fuel cells (SOFCs). This review discusses challenges, new findings and prospects for the implementation of these methods, with the main emphasis placed on the use of the ELD method. Topical issues concerning the formation of highly active SOFC electrodes using ELD, namely, the electrochemical introduction of metal cations into a porous electrode backbone, the formation of composite electrodes, and the electrochemical synthesis of perovskite-like electrode materials are considered. The review presents examples of the ELD formation of the composite electrodes based on porous platinum and silver, which retain high catalytic activity when used in the low-temperature range (400–650 °C). The features of the ELD/EPD co-deposition in the creation of nanostructured electrode layers comprising metal cations, ceramic nanoparticles, and carbon nanotubes, and the use of EPD to create oriented structures are also discussed. A separate subsection is devoted to the electrodeposition of CeO_2_-based film structures for barrier, protective and catalytic layers using cathodic and anodic ELD, as well as to the main research directions associated with the deposition of the SOFC electrolyte layers using the EPD method.

## 1. Introduction

The relevance of the application of electrodeposition in solid oxide fuel cell (SOFC) technology is associated with the search for new methods to increase the specific power and stability of the cells’ operation, particularly at reduced temperatures [1]. Enhancement of the cells’ efficiency using electrodeposition can be achieved by increasing the electrochemical activity of the electrodes through the deposition of catalytically active nanoparticles, reducing the cell ohmic resistance by the formation of a thin-film electrolyte membrane, blocking the leakage current or chemical interaction between functional layers by the deposition of thin buffer layers and by creating protective coatings on the interconnections [2,3,4,5,6]. The transition to the use of nanoscale heterostructure materials including two-dimensional materials such as graphene and its nanocomposites, metal–organic frameworks and metal oxide nanosheets, is a topical modern direction for increasing the efficiency of fuel cells operating in the low-temperature range (400–650 °C) [7,8,9,10,11]. Along with pulsed laser deposition and molecular beam epitaxy, allowing deposition of nanostructures without high temperature treatment, electrodeposition of nanocomposites—including those with graphene—are widely represented in technology for low temperature fuel cells as well as lithium-ion batteries, microbial fuel cells and supercapacitors [12,13,14]. Electrodeposition has been also demonstrated as a powerful technique to fabricate one-dimensional nanostructures such as nanorods, nanowires and nanotubes and composite films with controlled architectures [15,16,17,18].

Electrodeposition offers such advantages as being able to use simple technological schemes that do not require expensive vacuum equipment and the ability to precisely control the coating thickness by regulating the deposition modes. As this technique is based on the application of an electric field it is especially suitable for deposition on the substrates of complicated shapes, the formation of coatings on selected areas of the cells, the deposition of mono and multilayers, and impregnation of porous electrode substrates. Electrodeposition in SOFC technology can be considered not only as an independent technology, but also as an integral component in the context of a wide range of technological solutions, such as liquid chemical methods for the deposition of films from solutions, sputtering methods—magnetron sputtering, chemical vapor deposition (CVD) and pyrolytic deposition—as well as simple ceramic methods [19].

**Figure 1:**
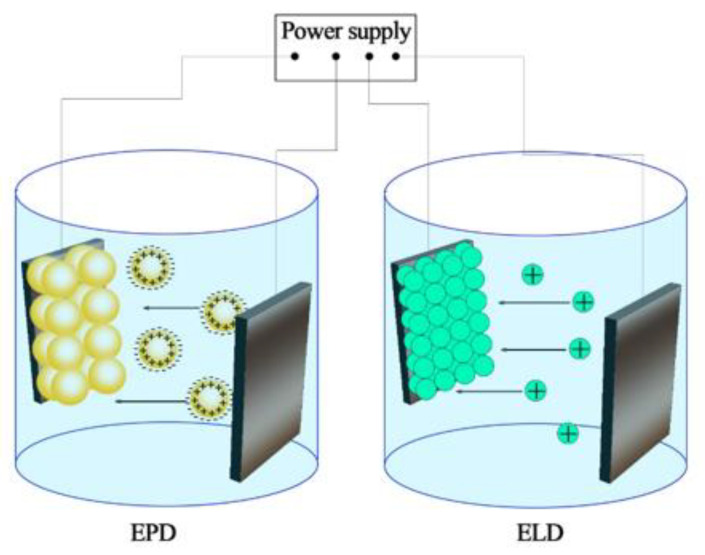
Schematic representation of the EPD and ELD processes.

Two variants of the electrodeposition processes have been developed for use in SOFC technology: electrophoretic deposition (EPD) and electrolytic deposition (ELD). The features of these two processes and their applications were analyzed in a number of reviews presented by I. Zhitomirsky [20,21]; the basic difference between these methods is that EPD is performed from suspensions of ceramic particles, while ELD is related to deposition from solutions of salts involving electrode reactions (Figure 1).

Metal ions or complexes are hydrolyzed by the electrogenerated base in the ELD process to form oxide, hydroxide or peroxide deposits on the substrates, which can be further converted to corresponding oxides by thermal treatment at relatively low temperatures. The formation of thin-film SOFC structures by the available ceramic and colloidal methods, including EPD, is in most cases associated with the high-temperature sintering of deposited coatings into dense ceramics, which causes difficulties in ensuring the compatibility of materials during their shrinkage under sintering and may be accompanied by the formation of cracks, pores, delamination of coatings, diffusion redistribution of metal cations and the formation of secondary non-conductive phases. In this sense, ELD, as well as co-deposition ELD/EPD technology, can be considered a way to expand the technological capabilities for obtaining functional SOFC layers, since the sintering of the obtained films is carried out at reduced temperatures—which eliminates a number of the aforementioned disadvantages. Compared to EPD, which can be applied to deposition films of different thicknesses and even compacts, ELD is preferable for the formation of various thin films—metallic, ceramic, polymeric and composite, with the opportunity to achieve smaller particle sizes and more uniform microstructures than those formed by EPD.

Recently, we presented a comprehensive review on the application of the EPD method in SOFC technology [22]. In this paper we demonstrate the possibilities and emerging problems in the formation of thin-film SOFC components using ELD; we direct our attention to studies on electrochemical synthesis techniques as cost-effective methods for obtaining thin film coatings based on prospective SOFC materials, the electrochemical introduction of metal cations into the porous structure of electrodes and options for the formation of thin-film electrolyte layers with a discussion on enhancing cell performance by using the electrodeposition technique. We also consider the use of ELD/EPD co-deposition in the creation of nanostructured coatings using metal cations, ceramic nanoparticles and carbon nanotubes. We also review a study concerning cathodic and anodic ELD and EPD of CeO_2_ films—topical for application both in SOFC technology and as highly active catalysts. Due to the lack of reviews on the application of ELD in SOFC technology we consider significant works published during the past two decades.

## 2. Electrodeposition Technology for the Fabrication of SOFC Air Electrodes with Increased Performance

Electrodeposition can be used as one of the stages in the formation of SOFC electrodes to increase their electrochemical activity, to decrease their polarization resistance and to increase their long-term stability under SOFC operating conditions. The electrochemical performance of an SOFC, especially at reduced operating temperatures, is mainly governed by the kinetics of the oxygen reduction reaction (ORR) at the air electrode (cathode). If the electrode comprises only an electronic conductor, the ORR occurs primarily at triple phase boundaries (TPBs) where the cathode catalyst, oxygen (gas) and electrolyte meet. In this case, in order to extend the TPBs, a composite electrode that comprises both an electronic conductor and an ionic conductor (solid state electrolyte) can be used. The composite electrodes can be made by mixing the components or by infiltration/impregnation. Two basic types of electrode structures can be obtained using infiltration/impregnation techniques: The first type consists of infiltrating an electronically conducting nanocatalyst (electrode material) into a single-phase ionic conducting backbone (electrolyte). The second type is related to infiltrating ionically conducting nanoparticles into a single-phase electronic conducting backbone. In addition, nanoparticles of the electrocatalyst, electrolyte and other oxides have also been infiltrated into mixed conducting backbones. In the case of a mixed electronic and ionic conducting cathode (MIEC) the ORR occurs at the surface of cathode particles and the resulting O^2−^ ions diffuse at the cathode/electrolyte interface. Thus, the TPBs are extended into the electrode volume. The interest in the formation of electrode films based on materials with a perovskite-like structure, which are mainly MIEC materials, is based on their potential use as oxygen electrodes in SOFCs and other electrochemical devices, as well as a high-temperature corrosion-resistant protective coating for interconnects. Screen printing [23], wet-powder spraying [24] and painting [25] are the most common methods used for the formation of SOFC cathodes. Numerous studies have shown that the electrode’s characteristics such as porosity, tortuosity and TPB length can be enhanced by modifying the cathode morphology and microstructure, thereby improving cathode performance. Methods such as electrospinning [26], plasma spraying [27], pulsed laser deposition [28,29], and chemical vapor deposition [30] have been successfully used to form nanostructured cathodes with high electrochemical characteristics. Methods of electrodeposition from solutions of metal salts and electrophoretic deposition of perovskite-like materials can be considered a cost-effective alternative to high-energy methods for obtaining highly active cathodes [31]. Also, the thickness of the deposited layers and the composition of functionally graded electrodes can be easily controlled in electrodeposition methods by varying the deposition time. For instance, using the EPD technique, Itagaki et al. fabricated bilayer SOFC cathodes, varying the thickness of the La_0.8_Sr_0.2_O_3−δ_ (LSM) and Zr_0.84_Y_0.16_O_2−δ_ (8YSZ) functional and LSM collector layers [32]. The developed electrode with an optimized thickness of the layers showed superior electrochemical activity (polarization resistance of 0.53 Ω cm^2^ at 600 °C) compared to those of the optimized single-layered cathodes (13.3 Ω cm^2^ and 1.3 Ω cm^2^ for LSM and LSM-8YSZ electrodes, respectively). 

### 2.1. Electrochemical Synthesis Techniques

Electrochemical synthesis techniques such as anodic oxidation, cathodic reduction and alternating current synthesis were developed as simple and cost-effective methods for obtaining thin film coatings, including those based on perovskite materials and seen as prospective for application as SOFC cathodes [19,33]. Sasaki et al. electrochemically synthesized perovskite materials such as LaMnO_3_ [34] and La_1−x_Sr_x_MnO_3_ [35]. It was revealed that even though La^3+^ions do not participate in oxidation reactions, they can be incorporated into anodically grown Mn oxyhydroxides through a specific adsorption of La^3+^ ions onto the surface of amorphous films. Single-phase perovskite manganite phases were obtained only when a large excess of [La^3+^] and [Sr^2+^] ions compared to Mn^2+^ ions (1000/1) existed in the electrolyte and the [La^3+^]/[Sr^2+^] ratio was equal to 1000/0 and 999/1. Heat treatment at 750 and 1000 °C was performed to obtain LaMnO_3_ and La_1−x_Sr_x_MnO_3_ coatings, respectively. All these experiments were carried out potentiostatically and the deposition conditions were determined empirically in a large number of experiments. The experiments performed for the LaNiO_3_ system in both galvanostatic and potentiostatic modes showed that La^3+^ ions strongly suppress NiOOH deposition. Heat treatment of the deposited film with a La/Ni atomic ratio at 600 °C led to the formation of a La_4_Ni_3_O_10.62_ perovskite-like layered phase but never to the formation of LaNiO_3_ perovskite [36]. The same scientific group proposed a preparation method of LaCoO_3_ perovskite using the similar procedure of anodic oxidation [37]. Aqueous solutions of Co(CH_3_COO)_2_, Co(NO_3_)_2_, CoSO_4_, and La(NO)_3_ were used as the electrolytes. The optimal [La^3+^]/[Co^2+^] ratio in the solution was found to be equal to 100. Single-phase LaCoO_3_ was obtained only at 0.95 V in the case of Co(CH_3_COO)_2_ solution and in the potential range of 0.70–0.95 V for CoSO_4_ and Co(NO_3_)_2_ solutions. These correspond to the formation of Co_2_O_3_ and the potential region above these values corresponds to the formation of CoO_2_. The presence of Co^3+^ ions, therefore, was found to be crucial for the incorporation of the La^3+^ in the solution. The precursor in this case was identified to be [Co_2_O_3_·2La(NO_3_)_3_·3H_2_O], which, after heat treatment at 500 °C, yielded the LaCoO_3_ coating. This temperature is significantly lower than that used for the calcination of LaCoO_3_ powders obtained by solid-state reaction (1000 °C [38]) hydrothermal synthesis (850 °C [39]) methods.

However, despite certain advantages of anodic oxidation, its main disadvantage is that La^3+^ ions have no anodic reactions of their own and their incorporation in the anodic deposit, as mentioned above, is achieved by enriching the bath with La^3+^ ions, and their consequent adsorption onto the surface of the amorphous metal oxyhydroxide films. This process does not allow complete control over the Ln^3+^ content in the coating. In order to overcome this problem, electrogeneration of the base by cathodic reduction of a mixed-metal (Ln^3+^+M^2+^/M^3+^) nitrate bath was proposed by Therese et al. for the synthesis of LaMnO_3_ [40] and perovskites of the LnMO_3_ (Ln = La, Pr, Nd; M = Al, Mn, Fe) and LaMO_3_ (M = Co and Ni) series [41]. It was demonstrated that nitrate reduction resulted in a steep increase in the pH value of the bath close to the electrode and facilitated the co-deposition of both the Ln^3+^ and M^2+/^M^3+^ ions; the resultant product is not simply a mixture of two hydroxides, but is an X-ray amorphous phase, demonstrating intimate interaction between respective hydroxides. It should be noted, that Mn^2+^ and Co^2+^ in the hydroxide precursors were oxidized to their trivalent states during deposition and the following thermal treatment, while oxidation of Ni^2+^ to Ni^3+^ was performed electrochemically by anodic polarization of the La,Ni-hydroxide coating in 6 M KOH at a current density of 1 mA cm^−2^ for 15 min [41]. The authors note that due to the difference in solubility of the products, the composition of the deposit varies from that of the bath, and so the bath composition was empirically optimized to obtain a single-phase perovskite oxide by thermal treatment performed in the temperature range from 550 °C (nickelates) and 675 °C (manganites) to 950 °C (cobaltites, aluminates and ferrites). The same scientific group obtained highly crystalline, very adherent LnCrO_3_ (Ln = La, Pr, Nd) coatings by the cathodic reduction of Ln(NO_3_)_3_ solutions followed by heat treatment of precursors at 750 °C [42]. Konno et al. [43] obtained La_1−x_M*_x_*CrO_3_ (M = Ca, Sr) thin films by cathodic reduction a mixed-metal nitrate solution containing (NH_4_)_2_Cr_2_O_7_ with the heat treatment at 700 °C.

Alternating current pulsing of the electrode in metal nitrate solutions improves the quality of the films and, therefore, was used in the preparation of many systems [33]. For example, Shi et al. [44] prepared doped manganese oxide coating electrodes with high electrocatalytic activity and stability by pulse anodic electrodeposition. The deposition rate was the highest when the pulse frequency was equal to 90 Hz and the pulse duty factor was 1:2. The oxide had a network structure that was a mixture of nanowires and spherical nanoparticles, which can effectively improve the electrocatalytic activity of the electrode. The service life of the electrodes was 1635 h, which was 55.3% higher than that of direct current electrodeposition. Intensification of processes of electrodeposition of metals by use of various modes of pulse electrolysis are considered by Kireev in a number of studies [45,46].

### 2.2. Fabrication of Composite Air Electrodes Based on Perovskite Electrode Materials Utilizing Electrodeposition Methods

The next stage in the development of film structures based on cathode materials using the electrodeposition method is the formation of composite electrodes. Electrodeposition can complement the known impregnation technology, widely used for expanding the TPB of the electrodes and improving contact on the electrode–electrolyte interface. The main disadvantage of the infiltration technique is that the process is tedious due to multiple infiltration and calcination steps [47]. The maximum amount of perovskite that can be added in a single step is equal to that which can be formed from the volume of liquid required to fill the pore volume of the backbone. Therefore, one step infiltration of 1 M solutions of La^+3^ and Mn^+3^ into a backbone of 65% porosity produces only 2.3 vol% of LaMnO_3_, while the required value is no less than 30%. Electrodeposition is expected to offer a powerful technique for fabricating composite SOFC electrodes with the required catalyst loadings in a single step [48,49]. Because electrodeposition can only be carried out on conductive surfaces, the porous backbone surface should be made electronically conductive before the electrodeposition. The composite LSM-8YSZ cathodes were prepared in [49] by electrodeposition of Mn^2+^ and La^3+^ into a porous 8YSZ substrate (65% of porosity). In order to make the 8YSZ backbone electronically conductive, prior to deposition the substrate was placed in flowing butane at 900 °C until 2 wt% of carbon was deposited in the pores. The Mn and La species were deposited one after another using 0.1 M solutions of their nitrate salts in dimethylsulfoxide (DMSO). All depositions were carried out galvanostatically at a current density of 4.5 mA cm^−2^. Once the deposition was complete, SrO was added by a single infiltration of Sr(NO_3_)_2_. According to SEM characterization performed after calcination at 900 °C, the deposits were in the form of small nanoparticles spread uniformly in the YSZ backbone, and the loading level of 33 wt% was reached. The crystalline LSM phase was formed after annealing at a temperature of 1100 °C. The electrochemical characteristics of the LSM cathodes obtained by electrodeposition and infiltration were shown to be identical. The ohmic losses of the electrodeposited cell at a temperature of 700 °C decreased from 0.65 Ω cm^2^ to ~0.55 Ω cm^2^ after short circuiting of the SOFC cell for 2 h, as a result of which, the I–V dependences became nearly linear, while for the impregnated cell they were 0.45 Ω cm^2^ and remained unchanged. The most prominent effect of shorting was on the polarization losses, which decreased from 2.1 to 0.6 Ω cm^2^ at 700 °C. Thus, electrodeposition can have certain advantages over impregnation in terms of the amount of material introduced in one deposition cycle, which will eliminate one of the disadvantages inherent in impregnation technology, which is the need to repeat a significant number of cycles to form a continuous spatial network of the introduced material in the porous structure of the supporting substrate. Zhao et al. and Pinto et al. selected for the formation of the composite air electrode MnCo_2_O_4_ (MCO) materials with spinel structures, traditionally used as a protective coating for ferritic stainless steels serving as interconnects for SOFCs [50,51]. Using alternating electrodeposition cycles of Mn and Co into a composite backbone of 8YSZ, the authors achieved MCO loadings of 35 and 43 wt% after four cycles of electrodeposition and subsequent annealing in air. Assuming that the MCO electrodes are limited by catalytic activity, a 40 wt% MCO-YSZ electrode was infiltrated with 5 wt% La_0.8_Sr_0.2_FeO_3−δ_ (LSF) to decrease its polarization resistance to an acceptable value of 0.3 Ω cm^2^ at 700 °C. Thus, MCO in these composite cathodes provides electronic conductivity and additional electrocatalysts must be introduced to provide enhanced cathode performance.

**Figure 2:**
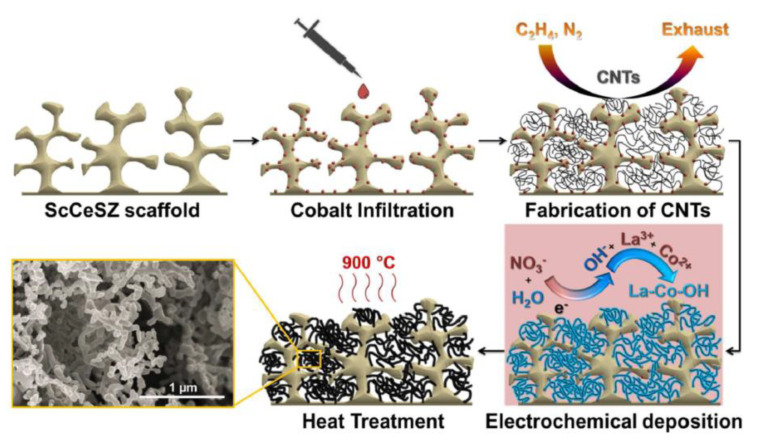
Schematic presentation of the electrode formation using the CAED method [52].

Chemically assisted electrodeposition (CAED) is a relatively new technique for fabricating nanostructured SOFC cathodes in a single loading step which has the advantage of the simultaneous deposition of multiple cations while using dilute aqueous solutions of salts. It was developed by Rehman et al. [52,53,54]. The proposed method involves CAED of mixed-metal hydroxide onto a carbon nanotube (CNT) template, followed by a low-temperature heat-treatment process (see the scheme in Figure 2).

**Figure 3:**
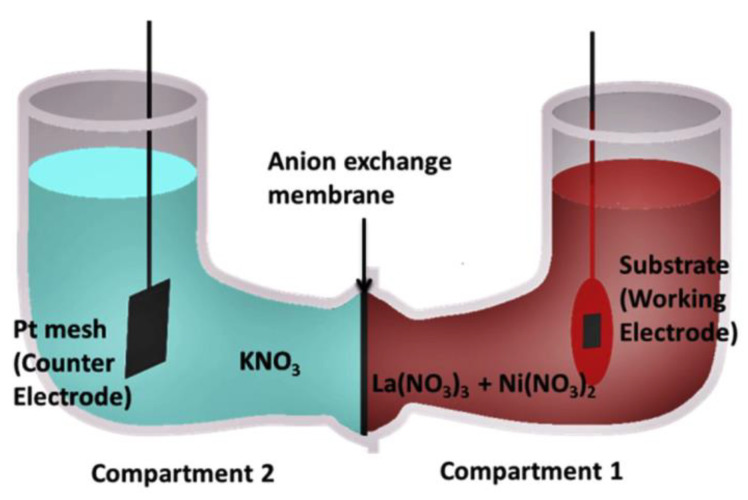
Schematic representation of the electrochemical cell used to perform the CAED experiments [53].

For the fabrication of composite cathodes by CAED, a two-compartment electro-chemical cell separated by an anion exchange membrane was used, as shown in Figure 3 [53]. Compartment 1 contained the mixed-metal nitrate solution; compartment 2 contained a KNO_3_ solution at the same concentration. CAED was performed galvanostatically by cathodic polarization of the working electrode with a current of approximately 1 mA cm^−2^. Nitrate ions (NO_3_^−^), having a higher value of the standard reduction potential as compared to metal ions, are reduced, preferably onto the working electrode, producing OH- ions. This leads to an increase in the pH value in the vicinity of the working electrode and, as a result, the metal ions present in the electrolyte form corresponding hydroxides and are precipitated onto the surface of the working electrode (CNT-modified porous electrolyte substrate).

In [52], the CAED method was applied to fabricate “nanofibrous” LaCO_3_ perovskites as cathode catalysts for SOFCs. To provide a catalyst for the growth of CNTs in the first step, a cobalt nitrate precursor solution was infiltrated into the porous (Sc_2_O_3_)_0.10_(CeO_2_)_0.01_(ZrO_2_)_0.89_ (ScCeSZ) scaffolds. After the cobalt infiltration, the CNTs were deposited onto the walls of the porous scaffolds by high-temperature catalytic chemical vapor deposition (CCVD). For that, the porous substrates were exposed to a flowing C_2_H_4_/N_2_ atmosphere inside a quartz tube reactor at 750 °C; by this method, the CNTs were grown on the cobalt nanoparticles during the decomposition of C_2_H_4_. In the next step, the LaCoO_3_ cathode was formed by the CAED method of electrodeposition from aqueous nitrate solutions at different La^3+^ and Co^2+^ ratios (1,1.5, 2, 2.5 and 3, followed by annealing in air at a temperature of 800 °C for 5 h. The authors showed that the anode-supported SOFC cell with a supporting anode, a thin-film ScCeSZ electrolyte (8 µm) and the developed nanofiber LaCoO_3_ cathode, containing 14.7 and 13.09 wt% of lanthanum and cobalt (at the ratio of cations in the solution 1:1), respectively, retained high strength and stability of operation at temperatures of 750 and 800 °C for 200 h at a galvanostatic load of 1 A cm^−2^. The ohmic resistance (0.47 to 0.30 Ω cm^2^) as well as the polarization resistance (1.11 to 0.90 Ω cm^2^) values of the SOFC with the nanofiber cathode decreased during 20 h of galvanostatic operation, due to the interface formation between cathode and electrolyte and the morphological changes in the cathode microstructure under loading, as is discussed elsewhere [55]. In the SOFC mode at a temperature of 800 °C, cell specific power densities of 811, 930, and 936 mW cm^−2^ were achieved after working for 24, 150, and 200 h, respectively. To obtain higher performance, an LaCoO_3_ nanofibrous cathode was prepared on a Ce_0.9_Gd_0.1_O_1.95_ (GDC) backbone. As a result, the cell specific power density was increased up to 1275 mW cm^−2^ at 800 °C, while the values of ohmic and polarization resistance were 0.18 and 0.61 Ω cm^2^, respectively; this is 2.65 times higher than the maximum power density of the SOFC with the conventional LaCoO_3_–ScCeSZ cathode sintered at 1200 °C, due to having to avoid the formation of insulating pyrochlore phases during the calcination of the electrode obtained by CAED at remarkably lower temperatures. In [53], CAED was applied to the fabrication of a cobalt-free LaNiO_3_(LNO)–Ce_0.9_Gd_0.1_O_1.95_ (GDC) composite cathode. The porous GDC backbone was fabricated by screen printing on the dense ScCeSZ electrolyte; then, CNTs were deposited onto the walls of the porous GDC scaffolds by the CCVD method [52]. For the fabrication of the LNO cathodes by CAED, a two-compartment electro-chemical cell separated by an anion-exchange membrane was used, as shown in Figure 3. Removal of the CNTs and formation of the LNO perovskite phase was revealed after heat treatment at 800 °C. The authors note that the proposed method, based on CAED technology, makes it possible to reduce the value of the polarization resistance of the electrodes to 1.632 Ω cm^2^ at a temperature of 650 °C. Although LNO possesses superior kinetics for ORR [56], it had not been previously used as an SOFC cathode, most likely due to its lower decomposition temperature (860 °C). Therefore, the application of CAED allowed the LNO electrode to be successfully employed as an SOFC cathode material.

**Figure 4:**
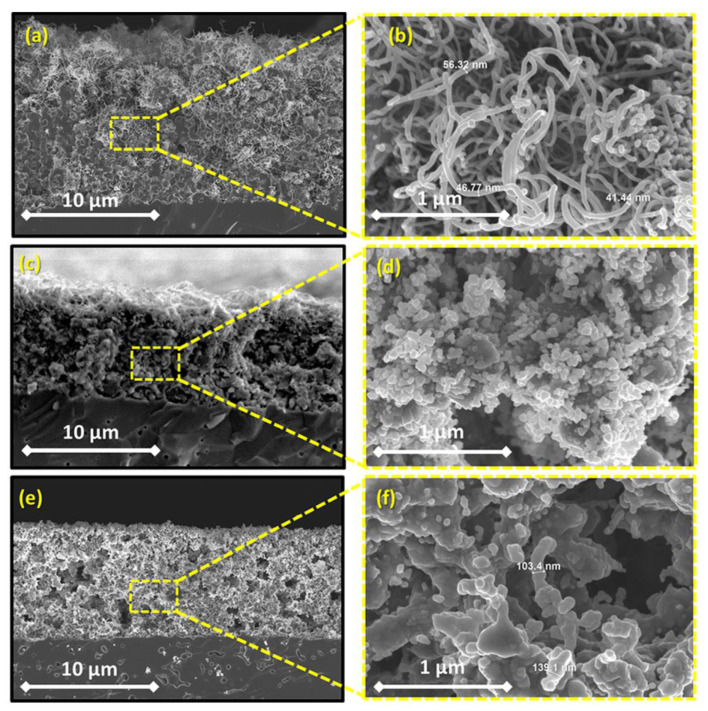
Cross-sectional SEM images illustrating the microstructural evolution during the fabrication of the n-LNF−GDC cathode: (**a**) CNT-modified GDC backbone, (**b**) magnified image of the CNTs, (**c**) Ni−Fe-coated GDC backbone, (**d**) magnified image of Ni−Fe coating, (**e**) nanofibrous n-LNF−GDC cathode annealed at 900 °C, and (**f**) magnified image of the n-LNF−GDC cathode [54].

Co-free LaNi_0.6_Fe_0.4_O_3−__δ_(LNF) perovskite has been reported as having high chemical stability in Cr-containing environments, high electronic conductivity and excellent electrochemical activity in SOFCs with composite air electrodes based on doped ceria [57,58,59]. However, LNF tends to react with zirconia-based electrolytes when prepared by conventional solid-state sintering at temperatures above 1000 °C with the formation of a La_2_Zr_2_O_7_ insulating layer and it is necessary to introduce a buffer layer to prevent the electrode–electrolyte interaction [59]. The sintering temperature can be significantly reduced using electrodeposition methods, including modified CAED. In [54], an integrated approach to the formation of an anode-supported SOFC with a thin-film ScCeSZ electrolyte (~5 µm) and a nanostructured LNF–gadolinium-doped ceria (GDC; n-LNF–GDC) cathode using various ceramic technologies (cold pressing, dip-coating, screen printing), as well as impregnation from solutions, CCVD, electroplating and CAED. The anode substrate was formed by isostatic pressing of a NiO–YSZ mixture (55:45) with 12 wt% pore former (graphite), followed by preliminary sintering at 1200 °C and applying a functional layer of NiO–ScCeSZ and a ScCeSZ electrolyte layer by dip-coating. Co-sintering of the NiO–ScCeSZ and ScCeSZ layers was carried out at a temperature of 1400 °C (3 h) in air. The GDC layer was formed on the electrolyte surface by screen printing using a slurry containing GDC particles dispersed in polymethyl methacrylate followed by sintering of the layer at a temperature of 1200 °C (3 h) in air. Cobalt nitrate was impregnated into the obtained porous GDC layer to create centers for the deposition of carbon nanotubes, which was carried out by CCVD with the decomposition of C_2_H_4_ in a mixture with nitrogen (50:50). Fe and Ni cations were co-deposited onto an electrically conductive CNT-modified GDC backbone by electroplating, whereas La cations were deposited using CAED as described above, as rare-earth elements cannot be successfully electroplated because of the extremely low values of their standard reduction potentials [60]. Subsequent annealing at 900 °C for 1 h formed a porous cathode LNF layer on the surface. The evolution of the electrode microstructure during its formation is shown in Figure 4. The LNF content in the cathode was found to be 20−25 wt%. The ohmic resistance of the obtained cell was 0.304, 0.293, and 0.281 Ω cm^2^ and polarization resistance was 0.415, 0.284 and 0.223 Ω cm^2^ at temperatures of 700, 750 and 800 °C, respectively. The maximum value of the power density was 984 and 1320 mW cm^−2^ at 750 and 800 °C, respectively. Polarization resistance of the developed n-LNF−GDC cathode was significantly lower than that for the LNF-GDC cathode fabricated by the authors using a conventional route (0.898 Ω cm^2^ at 750 °C), however, it was slightly higher than that of the LNF-SDC (samaria-doped ceria) conventional cathode with the optimized sintering conditions and electrolyte content of 50 wt% (0.15–0.21 Ω cm^2^ at 700 °C) [58]. Accordingly, this method may be further improved to achieve higher LNF loading. Long-term testing in a chromium-containing atmosphere showed excellent stability of both n-LNF−GDC and LNF-GDC electrodes compared to the Sr and Co-containing electrode.

### 2.3. Electrodeposition of the Composite Electrodes Based on Porous Pt and Ag

The key challenge in the development of low-temperature SOFCs that operate below 450 °C is the search for suitable cathode materials with the required combination of thermal and chemical stability, electrical conductivity and catalytic activity toward ORR. Traditional cathodes with perovskite structures such as (La,Sr)MnO_3_, (La,Sr)(Co,Fe)O_3_ and (Ba,Sr)(Co,Fe)O_3_ possess poor ORR activity in the LT range due to the high activation energy level of their polarization conductivity (1.5–2 eV). In this regard, platinum is viewed as an attractive candidate due to its process compatibility with micro-fabrication techniques, excellent ORR activity, and good electronic conductivity. To ensure a high site density of TPBs it is necessary to fabricate a highly porous but interconnected structure of the Pt electrode. However, upon sintering, porous nanoscale Pt films tend to grow into large, isolated crystallites, which results in a remarkable reduction of catalytic activity and current-collecting ability in the Pt electrodes [61]. To prevent Pt from agglomerating and extend the TPB site density, it was proposed to cover the surface of the Pt thin-film electrodes or infiltrate the porous Pt electrodes with 8YSZ [62,63] or doped ceria [64,65]. The chemical inertness of Pt minimizes possible interactions between infiltrated phases and the porous backbone structure.

**Figure 5:**
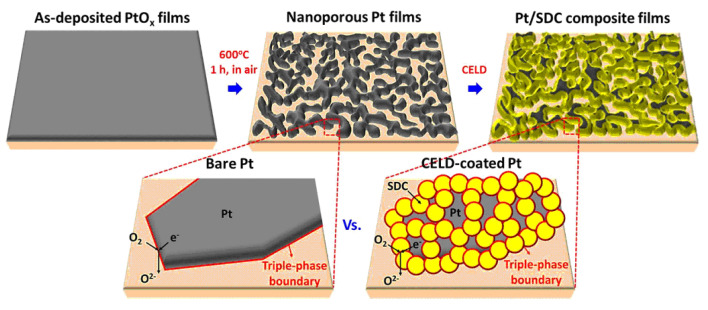
Schematic representation of the formation of a composite Pt-SDC film electrode and an increase in the length of the three-phase boundary [66].

Seo et al. [66] investigated the possibility of forming composite thin-film cathodes based on Pt particles using cathodic electrochemical deposition (CELD). The process of the formation of the Pt-Sm_0.16_Ce_0.84_O_2−δ_(SDC) composite cathode and the effect of increasing the length of the three-phase boundary are schematically shown in Figure 5. A YSZ single crystal (0.5 mm thick) was used as a carrier substrate, onto which a PtO_x_ layer (100 nm) was deposited by magnetron sputtering. The PtO_x_ coating was hardened upon cooling from 600 °C to room temperature, with the formation of a porous platinum layer as a result of thermal decomposition of the metastable PtO_x_ layer obtained by deposition. In the obtained porous Pt layer, SDC was electrochemically deposited from a solution of cerium and samarium nitrates saturated with oxygen in a potentiostatic mode at a potential of −0.7 V between the working electrode and the reference electrode. The SDC layer thicknesses were 21 nm and 76 nm with deposition times of 3 min and 5 min, respectively. Upon annealing the resulting structure at a temperature of 450 °C, the porosity of the SDC increased due to the development of nanosized cracks, resulting from a change in the specific volume of the cerium hydroxide during its oxidation. According to the authors, the cracks formed in the SDC layer improve the gas transport characteristics of the composite cathode. A CELD treatment of only 5 min created an oxide coating that increased the value of the electrode conductance by more than two orders. Cathode degradation studies were carried out at 600 °C for 100 h and showed a significant improvement in the thermal stability of the impedance of the composite electrode (Pt-SDC) compared to the virgin Pt electrode without the SDC coating. As a result, the authors have shown it is possible to improve the efficiency and stability of platinum cathodes by creating a composite core-shell structure.

Silver can be used as a low-temperature SOFC electrode and has a cost advantage over platinum, as well as excellent catalytic activity toward ORR and high electronic conductivity. To prevent particle agglomeration in a porous Ag layer, researchers considered the use of composite cathodes, in which Ag particles were coated with a layer of an oxygen-conducting electrolyte material. For this purpose, various technologies were applied such as aerosol-assisted chemical vapor deposition (AACVD) [67], atomic layer deposition (ALD) [68], sputtering [69], and infiltration [70].

The CELD method developed for deposition on Pt electrodes [66] was further extended by the authors to forming low-temperature Ag-based composite cathodes [71]. Cathodic electrodeposition of Pr_0.2_Ce_0.8_O_2−δ_ (PCO) film onto the surface of 400 nm-thick nanoporous Ag electrodes, formed on a single-crystal 8YSZ substrate by magnetron sputtering, was performed using a traditional three-electrode system with a standard calomel electrode for a reference electrode, a carbon rod as a counter electrode and a tailor-made aluminum clip connected with Ag electrode, which served as a working electrode. The sputtered Ag film had a columnar structure with nanoscale pores and very high TPB densities. The PCO film covered the Ag columns with flake-like particles. In the top-view image of the Ag–PCO film, micrometer-scale cracks were observed, as in the case of the Pt–SDC electrode [66]. It was shown that only 45 s of the CELD process resulted in 33-fold improved activity of the electrode (121.5 and 3.7 Ω cm^2^ at 450 °C). The reduction of the cathodic polarization resistance of the PCO-modified Ag electrode led to an increase in the peak power density of a single GDC electrolyte-supported cell (350 µm in thickness) and a porous Pt anode from 48.2 to 67.4 mW cm^−2^ at 450 °C. Consequently, CELD was proven to be a fast, simple, cost-effective, room-temperature deposition technique allowing ORR activity of the electrodes to be increased up to an acceptable level for low-temperature SOFC operation. Long-term measurements confirmed that CELD ensures superior thermal stability of Ag cathodes, allowing them to withstand use without any degradation, even at 550 °C for 50 h. The polarization resistance of PCO–Ag cathodes decreased down to 3.7 Ω cm^2^ (450 °C), while the polarization resistance on porous silver cathodes without coating was 121.5 Ω cm^2^.

### 2.4. Electrodeposition of the Electrodes with Pore-Formers and Development of Oriented Structures

One of the key challenges in SOFC technology is the formation of electrodes on a solid electrolyte surface with controlled thickness and porosity. Among electrodeposition methods, the application of EPD is preferable in this case, as ELD is suitable for the deposition of thin film, while the cathode thickness optimal for the majority of practical material choices and working conditions is in the range of 15–18 μm [72]. The EPD method affords the opportunity to deposit the electrode layers with high adhesive properties, which resolves the problem of layer delaminating and allows sintering of the electrodes at a low temperature, which decreases electrode/electrolyte interaction and preserves the electrode nanostructure [1]. The microstructure of the electrode films can be controlled by changing the applied voltage, the deposition time and the concentration of additives in the suspension. Additionally, to regulate the porosity of the EPD electrode, it is necessary to add pore-forming agents such as graphite or starch to the suspension based on the cathode.

**Figure 6:**
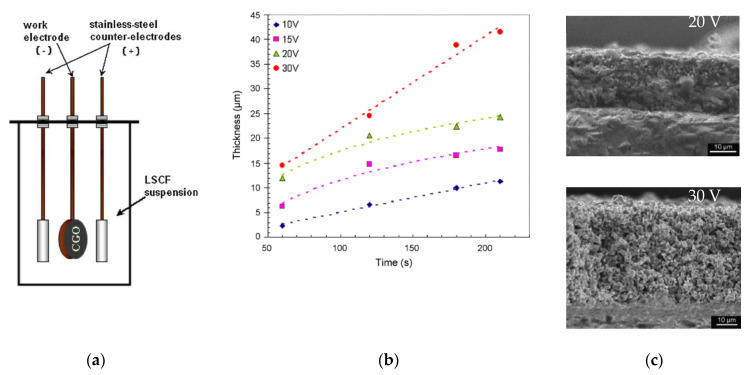
(**a**) Schematic diagram of the EPD cell used for the deposition of LSCF electrodes on both sides of the CGO disk substrate; (**b**) dependence of the deposit thickness on the deposition time for 1.05 wt% LSCF, 0.63 wt% iodine and 0.1 wt% starch in AcAc suspension at different applied voltages; (**c**) SEM images of the LSFC electrode with the same deposition weight deposited for 180 s at 20 V and 30 V [73].

Pioneering research on electrophoretic deposition of La_0.6_Sr_0.4_Co_0.8_Fe_0.2_O_3−δ_ (LSFC) cathodes on non-conducting CGO substrates was presented by the Boccaccini group [73]. Using a specific configuration of the EPD cell allowed deposition on both surfaces of the substrate to be performed simultaneously (Figure 6a). A comparative study of various suspension media (ethanol, acetone, acetylacetone (AcAc)) and pore-formers (starch and graphite) revealed that the optimal deposition rates were attained using a suspension comprising 1.05 wt% LSCF, 0.63 wt% iodine and 0.1 wt% starch in AcAc. It was shown that the thickness of the deposit increased appreciably with deposition time for the applied voltage of 30 V (Figure 6b) The increase in thickness at 30 V, compared with that obtained at 20 V at a similar deposition weight, clearly indicated an increase in the porosity of the deposited electrode layer (Figure 6c).

**Figure 7:**
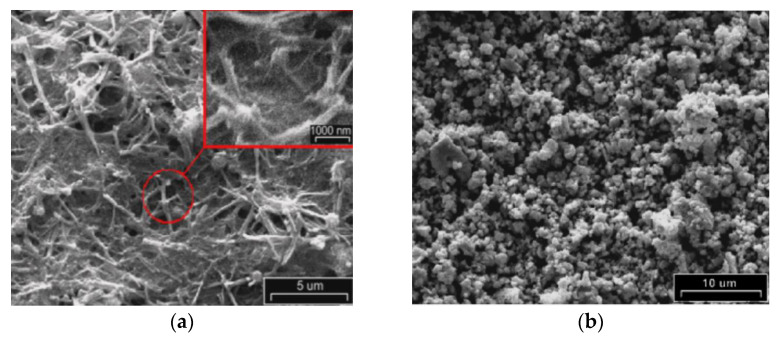
SEM images: (**a**) the surface of the CNT/LSCF composite film after preliminary annealing at 450 °C for 1 h; (**b**) the surface of the LSCF cathode after CNT burnout during annealing at 800 °C for 1 h [76].

It should be noted that the direct formation of a porous deposit of a cathode material by the EPD method on a non-conducting gas-tight solid electrolyte surface is impossible due to the absence of electronic conductivity on the electrolyte surface. This problem was solved by creating a conductive sublayer, for example, graphite [74], a conductive polymer—polypyrrole [75]—by means of surface metallization with Ag or Au [73,76], or by deposition of a Pt layer on the opposite side of the supporting electrolyte, which, after deposition, served as an anode during the cell operation [77]. The most complete review of the EPD of cathode materials, including that in micro-tubular cells, is presented in our recent review [22]. In the work of the Boccaccini group on the electrophoretic co-deposition of the cathode material LSCF and carbon nanotubes (CNT) on a supporting solid electrolyte [76], for instance, the conductivity of the GDC substrate, was provided by the deposition of an Ag sublayer. EPD was carried out from a composite CNT/LSCF suspension (in a wt. ratio of 1:10) in an acetylacetone medium supplemented with 2.45 wt% molecular iodine with respect to the concentration of the LSCF particles. A charging agent, iodine, added to the composite suspension, increases the effective charge on the particles due to the generation of protons in the suspension, which results in an increase in the deposited mass; this effect is discussed in a number of works on EPD [22]. The formation of a homogeneous coating required the selection of the voltage during the EPD; the optimal deposition mode was established at a voltage of 25 V for 4 min. Preliminary annealing at a temperature of 450 °C (1 h) showed the retention of carbon nanotubes in the structure of the deposited film, which were located parallel to its surface (Figure 7a). Upon final annealing in air at a relatively low temperature of 800 °C (1 h), the deposited carbon nanotubes played the role of a pore-former and burned out with the formation of a porous cathode structure with a thickness of up to 20 μm (Figure 7b). The authors note that the resulting electrode nano-microstructure will definitely enhance the cathodic performance, which is defined not only by the microstructure and porosity, but also by some degree of crystalline disorder around the electrode nanoparticles [78]. The sintering temperature of LSFC was further decreased in a study by Baharuddin et al. [79] by introducing 50 wt% of carbonate electrolyte based on samarium-doped ceria (SDCC) into the electrode composition. An EPD suspension based on a mixture of ethanol and deionized water with polydiallyldimethylammonium chloride (PDADMAC) was used as a dispersing agent. A symmetrical cell with LSFC-SDCC films deposited on the SDCC electrolyte and sintered at 600 °C showed a polarization resistance of 0.68 Ω cm^2^ at 650 °C.

Electrodeposition can be successfully applied to the deposition of the electrodes with oriented structure. It is an important issue as some layered materials (for example La_2_NiO_4_ (LN) with Ruddlesden–Popper structure) show significant enhancement of oxygen transport properties in the a-b plane. Matsuda et al. [75] fabricated an oriented LN cathode on a dense GDC electrolyte by electrophoretic deposition in an astatic magnetic field of 12T. The following EPD conditions were applied: voltage of 50 V, processing time of 5 min and 20 wt% starch added in the ethanol LN suspension, with the addition of phosphate ester and polyethylenamine as dispersants (2 wt% and 0.12 wt% to the LNO content, respectively). The cell performance with the a-b plane preferentially oriented LNO cathode was 1.5 times higher and polarization losses were 3 times lower than those in the randomly oriented one.

## 3. Electrodeposition Methods for the Formation of SOFC Ni-Cermet Anodes and Modification of Ni-Foam-Based Anode Collectors

Conventional SOFC anodes are prepared by mixing NiO and a solid-state electrolyte (Y or Sc stabilized zirconia (YSZ, ScSZ), SDC, GDC, protonic conductors based on doped BaCeO_3_–BaZrO_3_, etc.) followed by the formation of the anode layers (10–30 µm in thickness, single-layered or with a gradient structure) to the supporting electrolyte using such techniques as painting, tape casting, spin-coating or screen printing [80]. To achieve adequate electronic conductivity, large volumes of nickel (>30 vol% and typically 50 vol%) are needed. For the formation of an anode-supported half-cell comprising a thick supporting anode layer (typical NiO content 55–60 wt%, 300–700 µm in thickness), a functional anode layer (NiO content 45–50 wt%, 10–30 µm in thickness) and an electrolyte layer (10–30 µm), such methods as tape casting and tape calendering are used [81,82]. EPD was successfully applied to the formation of the anode layer on the electrolyte substrate [83,84,85], allowing comprehensive control over the layer thicknesses.

**Figure 8:**
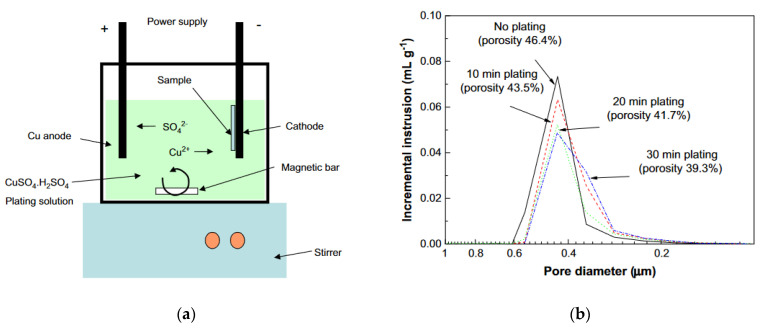
(**a**) The apparatus for Cu-electroplating; (**b**) the change in pore size distribution of Ni-YSZ anodes as a function of Cu-electroplating time [88].

As an alternative technique for SOFC anode formation, infiltration was used to introduce nanosized metal or oxide particles into a porous solid electrolyte backbone at relatively low temperatures, with the aim of engineering higher-performance electrode structures with an extended TPB length [86]. Also, the formation of porous electrolyte-tight, electrolyte-porous electrolyte structures following electrode impregnation is technologically easier and allows the production of large sized planar cells with excellent electrode–electrolyte contact and without deformation upon sintering. Infiltration enables the use of metals that cannot be processed at high temperatures, for example, Cu. It was shown that 9 vol% of infiltrated Ni was required to attain a percolated structure in the infiltrated electrodes [87]. However, at least 10 infiltration cycles were necessary to reach this value. Therefore, the practicability of upscaling this procedure is questionable. As in the case of composite cathodes, electrodeposition is considered a simple and fast method to produce composite anodes. Hyun at el. fabricated Cu–Ni–YSZ SOFC anodes for direct use of methane via Cu-electroplating [88]. The Ni–YSZ anode and the anode-supported single cell for Cu-electroplating were prepared by reducing NiO-based samples in H_2_ at 800 °C for 5 h. Cu/Ni weight ratios of 0.12, 0.38, 0.47 and 0.71 were obtained for 10 min, 30 min, 40 min and 60 min, respectively, by direct-current (DC = 0.1 A) electroplating the Ni–YSZ anodes with aqueous solution of CuSO_4_·5H_2_O and H_2_SO_4_. The scheme of the process is represented in Figure 8a.

The resulting samples were heated in H_2_ at 700 °C for 5 h. Before electroplating, the porosity of the Ni–YSZ anode was 46.4% and the range of pore distribution was 0.2–0.6 mm, while during electroplating it reduced down to 39.3% (Figure 8b), which is suitable for the SOFC anode. Carbon deposition tests have shown that the amount of carbon deposited on the Cu–Ni–YSZ anode (5.12–9.52 wt%) was from 30% to 50% less than that (14.83 wt%) deposited on the non-modified Ni–YSZ anode. The maximum power densities of the Cu–Ni–YSZ anode-supported cell were 0.32 W cm^−2^ in H_2_ and 0.24 W cm^−2^ in CH_4_ (at 700 °C) and decreased in the latter case to about 90% of the initial value for 200 h, while the Ni–YSZ anode-supported cell cracked after 21 h of testing in CH_4_ atmosphere.

The group of Corte et al. obtained bimetallic composites by electrodeposition of Cu into highly porous, 1.2 mm thick Ni–YSZ cermets and both Cr and Co into thin Cu–YSZ composites [89,90,91]. Later, this group demonstrated the opportunity to synthesize Ni, Co and Cu cermets by the electrodeposition of metals from an aqueous solution into a porous functional layer YSZ (thickness 60 μm, 65% porosity), preliminarily coated with carbon (11 vol% of carbon). The carrier substrate was a dense YSZ electrolyte (300 μm thick) [92]. Before the deposition, 10 vol% ceria was impregnated into the porous YSZ layer from aqueous solutions of Ce(NO_3_)_3_·6H_2_O followed by calcination at 450 °C in air. The authors note, that to achieve uniform plating of the metals inside the porous electrodes, the concentration gradients should be minimized by using low plating currents and relatively high concentrations of metal salts in the plating solution. It was demonstrated that up to 40 vol% of metal could be introduced into the porous YSZ backbone, while the carbon could then be removed by exposing the anode to humidified H_2_ at SOFC operating temperatures. As far as the authors were unable to prepare uniform Cu deposits directly onto the carbon-covered substrate, they successfully synthesized Cu–Co bimetallic composites by electroplating Cu onto Co. The performance of the cell with an anode containing 30 vol% Ni and 10 vol% ceria in humidified H_2_ at 800 °C was 149 mV cm^−2^ and decreased to 133 mW cm^−2^ after treatment to remove carbon (at 900 °C in 70% H_2_–30% H_2_O gas mixture); the cell with 40 vol% Co showed 175 mV cm^−2^ and 135 mW cm^−2^, respectively. The cell with the Cu–Co–ceria composite prepared with 15 vol% Cu electroplated onto 15 vol% Co exhibited the best performance of all the cells with a maximum power density of 175 m W cm^−2^ after carbon removal. Thus, in addition to providing an easy and scalable method for introducing metals into porous electrolyte backbones, the electrodeposition also allows preparation of novel layered electrode structures with superior performance, such as Cu–Co in [92].

**Figure 9:**
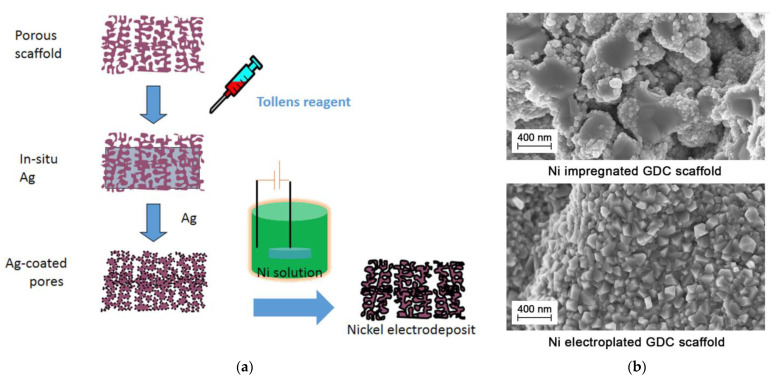
(**a**) Technological scheme of Ni ELD into the GDC electrolyte porous scaffold metallizing by silver using Tollens’ reaction; (**b**) SEM images of the GDC scaffold metalized with Ni using different methods [93].

Metallizing of the porous electrolyte scaffolds by Ag followed by impregnation of Ni particles by ELD was proposed by Ruiz-Trejo et al. [93] as a cost-effective fabrication method for solid oxide fuel cell anodes compared to the infiltration technique (Figure 9).

A layer of silver was deposited in the porous scaffolds using Tollens’ reaction: AgNO_3_ was dissolved in a solvent (2/3 water, 1/3 ethanol) and a few drops of concentrated NH_4_OH were added, until a black precipitate formed and then disappeared; next, KOH solution was added, resulting in the black precipitate reappearing; and then, enough NH_4_OH was added to re-dissolve the precipitate, leaving a clear solution. The GDC scaffolds, of 10 µm in thickness, fabricated by screen printing ceramic powder and pore-former mixtures onto YSZ discs followed by sintering, with an open porosity of ca 55%, were dipped into the solution. Finally, dextrose dissolved in the solvent were added to the mixture under stirring. The deposition of silver was finished within 5 min.

Ni was electrodeposited from a Watts bath with NiSO_4_, NiCl_2_ and H_3_BO_3_ and surfynol as a surfactant under a constant, but with an intermittent current (20 mA) to avoid starvation of Ni^2+^ inside the pores during deposition. The energy consumption for the electrodeposit used was minimal; with currents of 20 mA and voltages < 1 V, the power consumed was ca. 20 mW during deposits, while the conventional infiltration decomposition itself of nitrate to oxide requires repeated heating of the scaffold to at least 500 °C, with a typical furnace power consumption of kWs. The intermediate frequency resistance of the metallized electrode was 0.06 Ω cm^2^ at 600 °C in wet hydrogen and comparable with that (0.056 Ω cm^2^) obtained by the authors using standard impregnation methods [86]. However, the total area specific resistance (ASR) was relatively high and reached 1 Ω cm^2^ at 700 °C; it is probably related to a less-developed TPB length for the electroplated electrode (Figure 9b). The authors performed a series of studies for further optimization of the proposed method [94,95].

**Figure 10:**
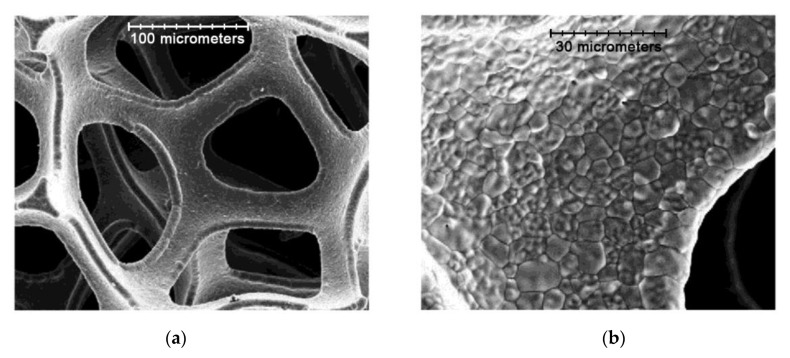
SEM images of Cu/SDC composite (9 vol% Cu) obtained by ELD on the Ni-foam. Scale bars: (**a**) 100 µm and (**b**) 30 µm [99].

Significant effort has been devoted to the development of anode catalysts for SOFCs fed by hydrocarbon fuels [96]. Ni-foam based substrates possessing high porosity, mechanical strength, good corrosion resistance and excellent electrical conductivity, mass produced by depositing nickel onto polymer foam followed by sintering it at high temperatures to remove polymer substrates, have been attracting increasing interest for their application as anode current collectors in intermediate-temperature SOFCs fueled by a wide range of natural and synthetic fuels. Open-cell metal foams made from Ni, Fe–Cr steel and Ni–Al intermetallide were shown to also be effective catalyst supports for internal indirect methane steam reforming in the intermediate temperature SOFC [97,98]. Surface modification of Ni foam can be performed using simple electrodeposition methods to increase its tolerance to carbon deposition and poisoning with H_2_S. Melnik et al. in [99] used two electrodeposition techniques to fabricate ceria ceramic and copper/ceria composite coatings onto Ni foil/foam substrates: cathodic electrophoretic deposition from ceramic GDC powder suspensions based on ethanol, and electrolytic deposition from aqueous solutions of CuSO_4_·5H_2_O and Ce(NO_3_)_3_·6H_2_O. Polyvinylbutyral-co-vinyl alcohol-co-vinyl acetate (PVB) and butoxyethyl acid phosphate (BAP) were used as a binder and dispersant for the EPD suspensions. GDC ceramic coatings prepared by EPD had a porosity of 20 vol% and formed structures which can be used as a porous matrix for SOFC composite anodes. Cu/GDC composite coatings with a higher content of ceramics in the Cu matrix (up to 20 vol%) were obtained in the framework of this study by being co-electrodeposited onto Ni foil or Ni foam at room temperature in an electrolytic bath containing an aqueous acidic solution of copper sulfate and a suspension of GDC nanopowder (SEM images are presented in Figure 10). It was shown that the composite Cu/GDC coatings with up to 12 vol% of GDC in the coating had high electrical conductivity (5.27 and 1.46 × 10^5^ S cm^−1^ at 20 °C and 600 °C, respectively)—comparable to that of Cu (5.96 and 1.76 × 10^5^ S cm^−1^). The ELD procedure developed in [99], was further applied by the same group to surface modification of Ni foam with Cu to use it as a coking resistant current collector for syngas (CO+H_2_ mixture, obtained by gasification of coal or steam reforming from natural gas)-fed SOFCs with a La_0.75_Sr_0.25_Cr_0.5_Mn_0.5_O_3−__δ_ (LSCM) anode catalyst [100,101]. Deposition was performed at room temperature and with a current density of 10 A cm^−2^. After the deposition, Cu-coated nickel foam was heat treated at 750 °C in H_2_. It was shown that the SOFC with surface-modified Ni foam provided approximately 10% higher specific power density than that with a gold mesh as a current collector (155 and 140 mW cm^−2^ testing at 900 °C) and demonstrated no significant degradation during 200 h. Cu-coated Ni foams also showed very stable resistance when using H_2_S containing syngas as a fuel. There was no formation of copper or nickel sulfide formation detected on the surface of the modified foam [101]. The authors note that Cu-modified nickel foam has a much lower cost compared to Au mesh current collectors used in syngas SOFCs, therefore, its usage can provide economic benefits for large-scale commercialization of such electrochemical devices. Direct electrodeposition is widely used for modification of Ni foam-based electrodes possessing low overpotential toward hydrogen and oxygen evolution reactions to improve efficiency of water splitting for hydrogen production [102,103,104].

## 4. Formation of Films Based on Cerium Dioxide by Electrodeposition

In this section, the studies discussed are on the preparation of films of doped and undoped CeO_2_ on conducting substrates by means of electrodeposition, which have the potential to be used both in SOFC technology, as electrolytes and composite electrode components, buffer electrolyte layers, catalytic layers or protective coatings for electrodes and interconnects, and in related fields—for example, the creation of highly active catalysts [8,105]. ELD allows coatings of atomic dimension to form and is suitable for the deposition of nanostructured coatings. In the electrolytic deposition of CeO_2_-based ceramic coatings, mainly aqueous solutions of salts are used; deposition of both thin-film and thick-film coatings is possible, however, there are limitations on the thickness of the resulting coating to 0.1–0.2 μm due to cracking of thicker coatings during consequent drying [106]. The EPD process is much faster than that of ELD, resulting in significantly higher deposit thicknesses and allowing the formation of the SOFC electrolyte films [107,108] and buffer cathode layers based on CeO_2_ over YSZ and LaGaO_3_ electrolytes [109,110].

### 4.1. Cathodic ELD

Under cathodic electrodeposition, the mechanism of CeO_2_ formation is based on a local increase in pH near the cathode during electrochemical reactions of water reduction with the participation of dissolved oxygen, the oxidation of cerium ions Ce^3+^ from nitrates with the participation of water to form Ce(OH)_2_^2+^, and the subsequent formation of CeO_2_:

2H_2_O + 2e^−^ → H_2_ + 2OH^−^ (1)

O_2_ + 2H_2_O + 4e^−^→ 4OH^−^ (2)

4Ce^3+^ + O_2_ + 4OH^−^ + 2H_2_O → 4Ce(OH)_2_^2+^ (3)

Ce(OH)_2_^2+^ + 2OH^−^ → CeO_2_ + 2H_2_O. (4)

The mechanisms of the cathodic reactions are discussed in [106,111]. Cathodic reduction of dissolved oxygen is described using various reactions, for example, by the direct reduction of oxygen with the participation of water to form hydroxide ions:

O_2_ + 2H_2_O + 4e^−^ → 4OH^−^, (5)

as well as by oxygen reduction with the participation of water to form hydrogen peroxide and hydroxyl ions:

O_2_ + 2H_2_O + 2e^−^ → H_2_O_2_ + 2OH^−^. (6)

The specific mechanism of oxygen reduction depends on the electrodes used, their electrode potential relative to a standard calomel electrode (SCE), the degree of oxygen saturation of the solution and the pH level of the solution [112].

Both CeCl_3_ and Ce(NO_3_)_3_ solutions were used to obtain CeO_2_-based coatings by cathodic ELD, however, more adherent and uniform coatings were obtained from chloride baths [113]. Zhitomirsky & Petric [114] studied cathodic electrodeposition of CeO_2_ and GDC films from aqueous and mixed ethyl alcohol–water solutions of CeCl_3_ and GdCl_3_ on Ni foil and Ni–8YSZ substrates. The influence of hydrogen peroxide on the electrodeposition process was studied. In the case of chloride baths, the hydroxyl ions are produced by the reduction of dissolved water (Equation (1)) and oxygen (Equation (6)). The addition of H_2_O_2_ leads to the formation of supplementary hydroxyl ions:

H_2_O_2_ + 2e^−^ → 2OH^−^ (7)

It was shown that H_2_O_2_ as a precursor prevented the formation of a non-stoichiometric cerium oxide film by oxidizing Ce^3+^ into Ce^4+^, the latter being more easily hydrolyzed. Also, the presence of H_2_O_2_ in the solution enhanced the adherence and uniformity of the deposited CeO_2_ films. Chloride ions can be partially removed from solutions containing H_2_O_2_ through an oxidoreduction process, diminishing the deposit contamination:

3H_2_O_2_ + 2HCl → 4H_2_O + O_2_ + Cl_2_ (8)

**Figure 11:**
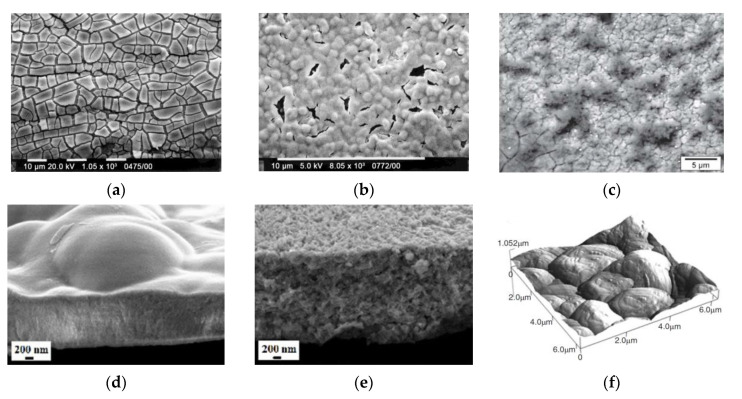
SEM images of the CeO_2_ deposit obtained from: (**a**) 0.01 M CeCl_3_ + 0.025 M H_2_O_2_ solution at a current density of 5 mA/cm^2^ [114]; (**b**) 0.01 MCeCl_3_ + 0.7 g/L PDDA solution at a current density of 3 mA/cm^2^ on a porous Ni-YSZ cermet substrate [114]; (**c**) 0.01 M CeCl_3_ in a water–ethanol mixture (1:9) at a current density of 0.25 mA/cm^2^ [115]; (**d**) 0.1 M NaNO_3_ + 5 × 10^−2^ M Ce(NO_3_)_3_ solution for 2 h at E = −0.8 V/SCE at 80 °C under argon bubbling [117]; (**e**) 0.1 M NaNO_3_ + 5 × 10^−2^ M Ce(NO_3_)_3_ solution for 2 h at E = −0.8 V/SCE at 30 °C under oxygen bubbling [117]; (**f**) AFM image of CeO_2_ deposited on a gold substrate from de-aerated 0.3 M CeCl_3_·7H_2_O solution [118].

As deposit-cracking during drying associated with shrinkage is a common problem for ELD coatings, in this study the authors also considered the formation of an organoceramic coating based on CeO_2_ by co-deposition with a polymer (polydiallyldimethylammonium chloride, PDDA), introduced into aqueous and mixed ethyl alcohol–water solutions. The cationic polyelectrolyte PDDA is a binder and its electrochemical incorporation (intercalation) inhibits the formation of cracks and improves the adhesion of the coating to the substrate. It was shown that cathodic deposition without a binder leads to the formation of cracks in the coating with a thickness of more than 0.2–0.3 µm during deposition both on a Ni foil and a porous Ni–YSZ cermet (Figure 11a). Also, by being electrodeposited on the porous substrate from CeCl_3_ solutions, the cerium species easily penetrated into the cermet anode and deposited on the inner surfaces of the porous network. When PDDA was used as an additive, penetration was blocked and the deposit formed on the top surface of the Ni–YSZ substrate. In the resulting coating with the PDDA binder, the number of cracks decreased; however, a continuous coating was obtained only by repeated deposition up to a total film thickness of about 2 μm (Figure 11b). The addition of PDDA reduced the deposited mass of the coating; consequently, the authors assumed that the co-deposition of the PDDA polyelectrolyte and CeO_2_ leads to shielding of the coating surface.

Creus et al. [115] studied the effect of the addition of a H_2_O_2_ precursor on the cathodic electrolytic deposition of CeO_2_ films on a steel substrate from a solution of CeCl_3_ in water and a water–ethanol mixture. The deposition was carried out in a classical three-electrode electrochemical cell with a calomel reference electrode. The counter electrode was a platinum grid. The deposition was carried out in a galvanostatic mode without stirring at room temperature. It was shown that the addition of hydrogen peroxide to an aqueous solution provided oxidation of cerium ions and growth of deposited CeO_2_ films, while electrodeposition in the aqueous solution without H_2_O_2_ was accompanied by the formation of hydrogen, which prevented the film formation. In a solution based on a water-ethanol mixture, the electrolytic deposition of CeO_2_ films occurred without the H_2_O_2_ addition. The morphology of the films deposited from the solutions with the addition of H_2_O_2_ depended on the exposure time of the solution before the deposition, which was accompanied by a change in the pH value and, as a consequence, led to the irreproducibility of macroscopic defects in the film and its composition. As a mechanism for the growth of CeO_2_ films during their electrolytic deposition, one can distinguish the nucleation of individual crystallites on the substrate surface, which appear in the gel layer on the electrode surface and become active centers for further growth of the deposit [116]. In this case, the deposited films have a loose structure with a significant number of cracks and pores (Figure 11c), as was observed by Creus et al. [115]. Dark spots at the surface of the oxide coating were identified as ferrihydrite. This corrosion product, which appears during drying, partially seals the cracks and defects of the oxide film.

In addition to the above-described mechanism of cathodic deposition with the participation of dissolved oxygen according to reactions (3) and (4), Dronova et al. [117] proposed a mechanism for the cathodic deposition of CeO_2_ without the participation of dissolved oxygen under the conditions of pumping argon through a solution containing 0.1 M of NaNO_3_ and 5 × 10^−2^ M of Ce(NO_3_)_3_, heated to 80 °C. According to the authors, nitrate ions are reduced to hydroxide with an increase in pH at the cathode, then the formation of cerium hydroxides and their oxidation to cerium dioxide occurs:

NO_3_^−^ + H_2_O + 2e^−^ → NO_2_^−^ + 2OH^−^ (9)

Ce^3+^ + 3OH^−^ → Ce(OH)_3_ (10)

Ce(OH)_3_ + OH^−^ → CeO_2_ + H_2_O + e^−^ (11)

The resulting CeO_2_ films obtained under argon bubbling are thinner (up to 1 μm) and have a denser structure (Figure 11d) compared to those obtained under oxygen bubbling at room temperature (Figure 11e) with a thickness of 1.5 μm.

The kinetics of cathodic electrodeposition of Ce^3+^ and/or Ce^4+^ oxides from aerated and inert alcoholic electrolytes on gold substrates was studied by Valov et al. [118]. For the aerated solution, a direct electrochemical reduction of positively charged Ce-oxygen complexes to Ce_2_O_3_ was suggested, while for de-aerated electrolytes cerium was supposed to deposit in the metal state being oxidized to a CeO_2_ upon exposure to air. In the latter case, a classical spiral crystal growth, typical for metals, was observed (Figure 11f) and the film structure differed considerably from that of Ce_2_O_3_.

**Figure 12:**
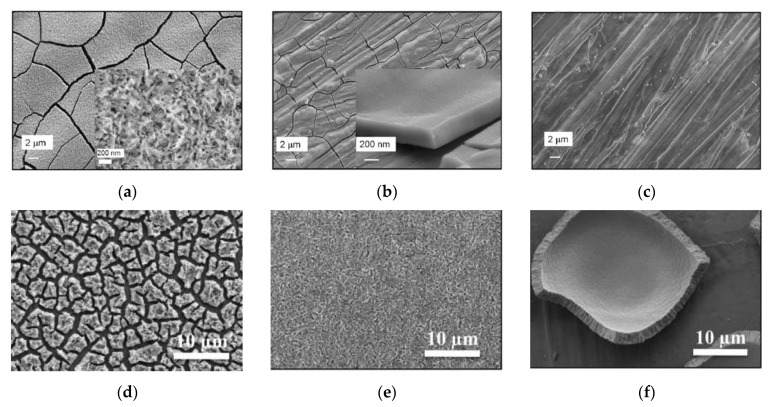
SEM images of the surface as-prepared films deposited potentiostatically at E = −0.8 V/SCE potential at 30 °C on stainless steel substrates: (**a**) SDC0; (**b**) SDC02; (**c**) SDC30; (**d**) CeO_2_; (**e**) Gd_2_O_3_; (**f**) GDC23 [120,121].

**Figure 13:**
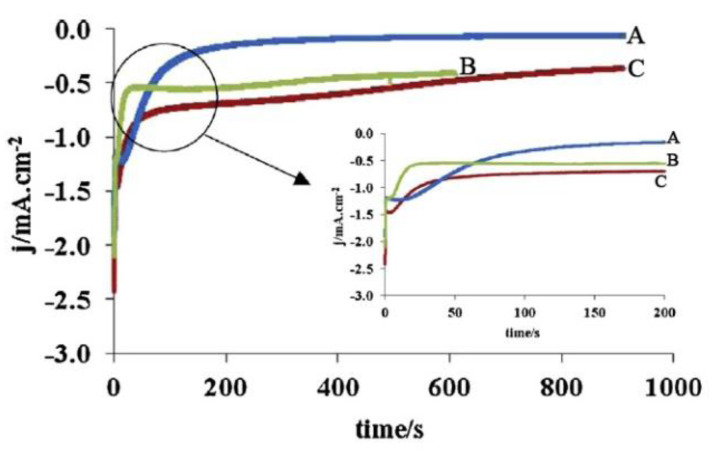
Chronoamperograms obtained during deposition of Gd_2_O_3_, CeO_2_, and GDC from 0.005 M of Gd^3+^ (A curve), 0.005 M of Ce^3+^ (B curve) and mixed 0.005 Ce^3+^/0.0005Gd^3+^ (C curve) solutions [121].

Lair et al. [119,120] conducted a study on the effect of the addition of samarium over a wide range of concentrations (0–50 at%) on the properties of thin films of ceria electrodeposited potentiostatically on a 316L stainless steel substrate. It was shown that an increase of the Sm content up to 1 at% (SDC01) induced an enhancement of the CeO_2_-based phase crystallinity, while with further increases in the Sm content from 1.0 to 1.5 at% the intensity of CeO_2_ peaks decreased; 2 to 30 at% of the Sm content (111) and (200) diffraction peaks could not be observed in XRD patterns, indicating the appearance of amorphous structures. After annealing at 600 °C the slight appearance of a diffraction line, corresponding to the (111) peak was revealed, suggesting the formation of SDC crystallites in Sm-rich films. The SDC films were more adherent and less cracked in comparison to those of CeO_2_. With increasing Sm content over 2 at%, the deposited films became more compact, with a smooth, gel-like surface. The SDC30 film possessed a glass-like morphology without visible grains. Evolution of the film structure dependent on Sm content is shown in Figure 12a–c [120]. The crystal quality of the films, however, was depressed by increases in Sm-doping atoms, e.g., for 50 at% [119]. The same group represented one-step cathodic electrodeposition (from the common solution bath) of Gd-doped ceria films [121]. CeO_2_, Gd_2_O_3_ and Ce_0.77_Gd_0.23_O_2−δ_ (GDC) thin films were deposited at room temperature applying a fixed potential value of −0.8 V vs. SCE potential. The supporting electrolyte was 0.1 M NaNO_3_, in which metal nitrates were added. SEM observations of the films deposited during 15 min and annealed at 600 °C revealed that the gadolinia films were more uniform and less cracked compared to ceria and GDC films (Figure 12d–f). The GDC film was flake-like with the highest deposit thickness of 1250 nm (160 nm and 600 nm for gadolinia and ceria films). The authors note, however, that GDC films are more compact, even if having mesoporous morphology. The authors discuss the features in the current density change with time during electrodeposition of CeO_2_, Gd_2_O_3_ and GDC films. The experimentally obtained chronoamperograms indicate a different mechanism for the formation of Gd_2_O_3_ films. For all curves (Figure 13A–C) it is noted that the current density sharply decreases in the first 100–200 s, then reaches saturation (a plateau). However, for CeO_2_ and GDC, the current density value first sharply decreases (in the first 2–3 s), a small peak appears before the next decrease, then current density reaches saturation and, in the authors’ opinion, this form of chronoamperogram is explained by the nucleation and growth of a non-conducting oxide film. The chronoamperogram for pure Gd_2_O_3_ looks somewhat different: in the first few seconds the current density rapidly decreases and then, after 20–30 s, reaches the plateau, which the authors associate with the formation of Gd(OH)_3_, then the current density decreases again and reaches saturation (the second plateau for this curve), which may already be associated with the formation of a film of pure Gd_2_O_3_. It is important to note that, in order to obtain a homogeneous coating without cracks, it is necessary that the final current density tends to zero—for the Gd_2_O_3_ film this condition is fulfilled (0.06 mA/cm^2^) and it agrees well with the microstructural data (Figure 12e). The same group from France applied the developed technology of electrodeposition to obtain 1-D ceria-based structures [122]. Cathodic deposition was performed in the potentiostatic mode at a fixed potential value of −0.8 V vs. SCE from the electrolytic bath containing 2.5 mM Ce(NO_3_)_3_ at 30 V for 20 min at room temperature on the F-doped tin oxide (FTO) film and indium-tin-oxide (ITO) film-coated glass substrates. The formation of randomly orientated ceria nanorods of about 85 nm and a length of up to 700 nm, was observed on the FTO substrate, while a nanocolumnar dense film characterized by the presence of hexagonal-spherical particles of about 150 nm in radius 1 was grown on the ITO substrate. The annealing at 250–600 °C was found to enhance crystallinity and crystal quality of the deposited films.

Phok & Bhattacharya [123] obtained CeO_2−δ_ and Ce_1−x_Sm_x_O_2−δ_ films (x = 0.08 and 0.2) with a thickness of about 100 nm on Ni–W substrates. The films were deposited at a current density of 0.17 mA cm^−2^ from a solution of cerium and samarium halides dissolved in deionized water at an electrolyte pH of 4. The average deposition rate was 10 nm/min. The deposited films were either amorphous or polycrystalline with a predominantly biaxial texture after annealing at a temperature of 910–980 °C for 2 h in an Ar/0.5% H_2_ mixture. The crystallite size of the Ce_0.8_Sm_0.2_O_2−δ_ (SDC20) increased with an increasing temperature from 18 nm at 910 °C to 35 nm at 980 °C. At an annealing temperature of 940 °C, no cracks were observed in the SDC20 coating, in contrast to the films annealed at higher temperatures. Films of undoped CeO_2_ were characterized by a higher crystalline size of 52 nm and the formation of cracks. The authors assume a significant influence of the crystallite size on the formation of cracks in the coating, namely, that with an increase in the crystallite size above the critical value, the influence of mechanical stresses in the film increases, which leads to the nucleation and growth of a network of cracks.

### 4.2. Anodic ELD

**Figure 14:**
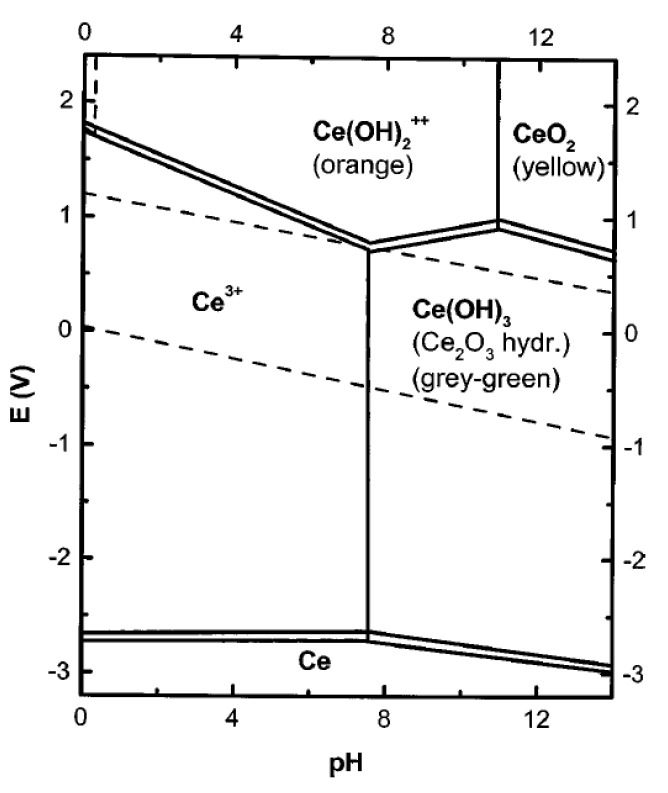
A simplified Pourbaix diagram of cerium species [124,125].

For anodic deposition, two methods for the oxide film formation can be considered. One method involves the oxidation of a metal electrode by metal oxide formation and the other is related to the oxidation of species in the solution from a lower oxidation state to a higher oxidation state [124]. A simplified Pourbaix diagram (E vs. pH) for cerium species, showing redox species and solubility products in an aqueous solution is shown in Figure 14 [124,125]. According to the diagram, a possible mechanism of anodic deposition of CeO_2_ from an aqueous solution was proposed by Golden & Wang [124]. Below pH = 7, Ce^3+^ ions are stable between the reduction and oxidation limits of the electrolyte (dotted lines on the diagram). At voltages above ~1 V, a hydroxide species is formed in the solution as follows:

Ce^3+^ + 2H_2_O → Ce(OH)_2_^++^ + 2H^+^ + e^−^, (12)

which can undergo a chemical reaction with the formation of CeO_2_ in the solution:

Ce(OH)_2_^++^ → CeO_2_ + 2H^+^. (13)

If the pH of the solution is above 7, then an insoluble precipitate of Ce(OH)_3_ is formed. When Ce^3+^ ions are stabilized in a solution by the addition of organic acids, for example, acetic acid, lactic acid, citric acid, oxalic acid and EDTA [124,126], the formation of Ce_2_O_3_ oxide is possible:

2Ce(III)-L → 2Ce^3+^ + 2L → 2Ce^3+^ + 3H_2_O → Ce_2_O_3_ + 6H^+^, (14)

which can then be oxidized to form an anodically deposited CeO_2_ film:

Ce_2_O_3_ + 2H_2_O → 2CeO_2_ + 2H^+^ + 2e^−^. (15)

Balasubramanian et al. [126] revealed, by using an X-ray absorption study of its local structure, that cerium was present in the 4^+^ oxidation state in films prepared by anodic deposition from 0.08 M cerium (III) acetate solution at 1.1 V/SCE. In the films, prepared by cathodic deposition from 0.05 M Ce(NO_3_)_3_ solution at a cathodic current of 2.5 mA cm^−2^, contrarily, cerium was predominantly in the 3+ oxidation state with a small amount of Ce^4+^ ions [126]. The local structural and electronic order of cerium in the anodic films was found to be more similar to that of Ce(OH)_4_ than to CeO_2_. This shows that the bonds in the anodic films are highly structurally disordered when compared to CeO_2_. The result is the formation of a coating with a vitreous non-crystalline structure, acting as a superior passive film against corrosion.

**Figure 15:**
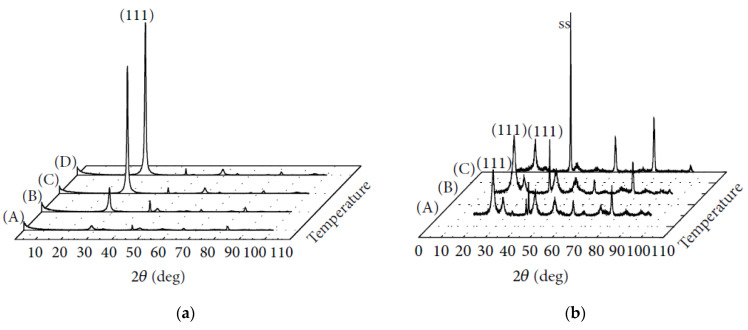
XRD patterns of CeO_2_ films deposited from a solution of 0.1M Ce(NO_3_)_3_ and 0.1M acetic acid at pH = 7.5: (**a**) in a galvanostatic mode at a current density of −0.06 mA cm^−2^ at a temperature of (A) 25, (B) 50, (C) 70, and (D) 80 °C; (**b**) in a potentiostatic mode at a deposition potential of 1.10 V at a temperature of (A) 25, (B) 50, and (C) 70 °C [128].

To establish the optimal conditions for the anodic deposition of CeO_2_ films using a ligand for the stabilization of Ce^3+^ ions in the solution (Equations (15) and (16)), a systematic study of the experimental parameters was performed by Wang & Golden [127,128]. Deposition was carried out on stainless steel at a voltage of 1–1.2 V for 24–48 h from a 0.1 M aqueous solution of Ce(NO_3_)_3_·6H_2_O, with the addition of acetic acid as a complexing agent and NaOH to adjust the pH value. Predominant bidentate binding of the cerium ion with the formation of a [Ce(H_2_O)_x_(O_2_CCH_3_)]^2+^ species (the coordination number of the Ce(ΙΙΙ)-acetate complex was estimated to be equal to 6–7) was observed for the 1:1 complex and bridging at higher acetic acid concentrations, which is in agreement with the results obtained in [129,130]. In a galvanostatic mode, with a change in the deposition current from −0.90 to −0.06 mA cm^−2^ the films deposited at 25 °C exhibited more oriented XRD patterns and the intensity of the (111) reflection increased. When increasing the deposition temperature at the constant current of −0.06 mA cm^−2^, the intensity of the CeO_2_ (111) reflection increased, indicating the deposition of the films with a preferred (111) orientation (Figure 15a) [128]. At the same time, the films deposited in a potentiostatic mode possessed a random structure regardless of the deposition temperature (Figure 15b). The estimated crystallite size for the preferred oriented CeO_2_ films increased from ∼10 nm for the green film to ∼120 nm for that sintered at 900 °C. SEM characterization revealed a smooth surface and the appearance of cracks in the deposits, which became more extended when increasing the sintering temperature.

Kulp et al. [129], obtained CeO_2_ thin-film coatings using anodic deposition from an aqueous solution of cerium nitrate with the addition of ammonium acetate to stabilize the Ce^3+^ ions. It was shown that at the applied anode potential, with respect to the reference electrode of +0.5 V and pH ~ 6.1, direct oxidation of Ce^3+^ ions occurred with the formation of a smooth, crack-free CeO_2_ thin-film coating. With a deposition time of 1000 s, the coating thickness was about 40 nm. The authors noted that at a higher anode potential (+1.1 V), water was electrochemically oxidized during the deposition with the generation of oxygen which reacted with the Ce^3+^ ions, and that the formation of CeO_2_ occurred far from the electrode. In this case, the resulting coating exhibited cracks and was weakly bonded to the electrode.

**Figure 16:**
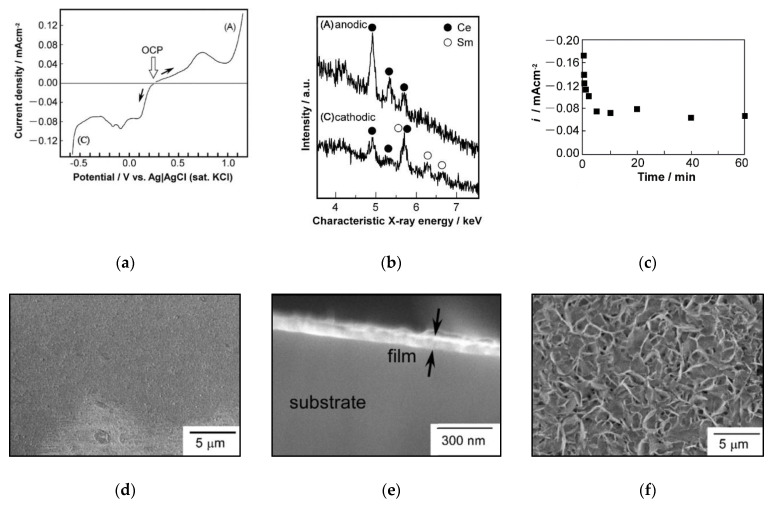
(**a**) Anodic (A) and cathodic (C) polarization curves (2 mV/s) of a Pt electrode obtained in the mixed-metal solution (Sm content of 0.5 mol%); (**b**) EDS spectra of the films deposited by anodic (+1.0 V) and cathodic (−0.5 V) polarizations; (**c**) Chronoamperometry curve of the SDC film growth on the Pt electrode at −0.5 V; (**d**,**e**) the surface and cross-sectional SEM images of the SDC film deposited with acetic acid; (**f**) the surface image of the SDC film deposited without acetic acid [131].

A comparative study of cathodic and anodic deposition was carried out by Kamada et al. [131], in which the formation of a thin-film layer of Sm-doped CeO_2_ was performed using both anodic and cathodic deposition from an aqueous 0.1 M solution Ce(NO_3_)_3_ + Sm(NO_3_)_3_ with the addition of 0.1 M acetic acid. The fraction of Sm^3+^ ions was in the range 0–0.5 mol%. To ensure the conditions for the dissociation of acetic acid and complexation between acetate ions and Ce^3+^ ions, the pH value was adjusted to a value of about 5 by adding NaOH. As a result of experiments, it was shown that the occurrence of cathodic or anodic deposition depends on the potential of the Pt electrode relative to the reference electrode (Ag, AgC|Cl^−^; Figure 16a); anodic deposition occurred at a potential higher than +0.8 V, and cathodic deposition at a potential of lower than −0.5 V. During anodic deposition, the resulting film, according to the data of energy dispersive analysis, did not contain Sm, while during cathodic deposition, the Ce- and Sm-containing films were co-deposited (Figure 16b); therefore, the authors concentrated further work on optimizing cathodic deposition to obtain an SDC film. At potentials of the working electrode more positive than −0.4 V, no deposition took place, whereas at potentials lower than −0.8 V, a thick opaque deposit was formed, weakly adhering to the substrate. Therefore, the authors chose the optimal value of the potential of the working electrode, which was equal to −0.5 V.

The corresponding change in current with time is shown in Figure 16c, which shows the initial drop in current and the subsequent attaining of a stationary value of ~0.08 mA cm^−2^ in approximately 5 min, which, as noted by the authors, indicates a slowdown in electrode reactions due to the formation of an insulating layer over the entire surface of the working electrode. The authors revealed formation of Ce(OH)_3_ from Ce(Ac)^2+^ complexes on the Pt electrode surface during electrolysis by the following chemical reaction:

Ce(Ac)^2+^ + 3OH^−^ → Ce(OH)_3_ + Ac^−^ (16)

The morphology of the SDC film with a thickness of ~100 nm obtained with the addition of acetic acid was uniform, did not contain cracks or breaks and had an average crystallite size of about 10 nm. However, deposition without the addition of acetic acid led to the formation of deposits in the form of individual plates separated by cracks. The mechanism of the influence of the addition of acetic acid on the deposition, according to the authors, is associated with the process of complexation of Ce^3+^ ions with acetate ions and the gradual growth of the deposited layer of cerium hydroxide. The authors also see a possible effect on the uniformity of the coating in the relaxation of the pH gradient near the working electrode due to buffer action of the free acetate ions (Equation (16)), which results in the formation of a precipitate of cerium hydroxide closer to the electrode surface; subsequently, cerium hydroxide is successfully converted into a CeO_2_ precipitate on the electrode.

### 4.3. Electrophoretic Deposition of CeO_2_-Based Thin-Film Electrolyte Membranes

One of the main requirements for SOFC solid electrolyte membranes is that of absolute gas-tightness. In addition, there is a need to obtain membranes based on multielement solid solutions and composite electrolyte membranes. For SOFCs with a MIEC solid electrolyte membrane based on doped CeO_2_, which displays mixed conductivity in a reducing atmosphere, it is necessary to form the electrolyte layers with a thickness of at least 5 microns or more in order to reduce the effect of the electron current short circuit on the cell efficiency and to form barrier layers to block the electronic current on the anode or cathode side. Dense CeO_2_ barrier layers, in their turn, are used to prevent chemical interactions between the ZrO_2_ and LaGaO_3_-based electrolytes and the SOFC electrodes [8]. The use of EPD in the technology of creating SOFC film electrolyte membranes has advantages over ELD in terms of a higher deposition rate and the possibility of obtaining micron-thick films, including multilayer electrolyte films with increased adhesion of the layers [132]— and it also allows deposition of the whole SOFC structure [133]. Electrophoretic deposition is performed from a suspension of ceramic particles of a given composition (Figure 1), or possibly, a multicomponent composition. The formed film has the same composition [107] which distinguishes the EPD method from the ELD method—the implementation of which is associated with the synthesis of oxide material from salt solutions on a conducting substrate and in which there are difficulties in obtaining even two-component solid solutions of a given composition [131]. In this subsection, works related to the application of the EPD method to obtain thin-film electrolyte membranes based on doped CeO_2_ for intermediate and low-temperature SOFCs, including microtubular ones, are discussed.

A single-layer SDC electrolyte was obtained by Ichiboshi et al. [134] by EPD from a suspension of SDC powder in ethanol with the addition of iodine (0.6 g L^−1^). The size of the SDC particles in the dispersion medium was ~0.4 μm. The resulting coating after sintering at a temperature of 1600 °C (10 h) had a thickness of ~20 μm and was characterized by the presence of cracks due to the different shrinkage characteristics of the electrolyte layer and the Ni-SDC anode substrate. At a temperature of 700 °C, the single SOFC had a specific power of 60.6 mW cm^−^^2^ at an OCV value of 0.63 V. In this work [135], the sintering of a single-layer EPD-deposited SDC electrolyte was carried out at a temperature reduced to 1400 °C (25 h). EPD was carried out on a NiO-SDC substrate with a particle size of 49 and 193 nm, with the addition of a pore former (carbon) and a binder (PVB). Acetylacetone with the addition of iodine (0.5 g L^−1^) was used as a dispersion medium for EPD. A sintered SDC electrolyte layer of 13 μm in thickness was gas-tight and crack-free. This was achieved by selecting an appropriate amount of pore-forming agent and particle size in the composition of the Ni–SDC anode substrate, which made it possible to control the degree of substrate shrinkage. A specific power of 272 mW cm^−^^2^ and an OCV value of ~0.65 V were attained at a temperature of 700 °C. Yu et al. [136] demonstrated the possibility of creating the entire SOFC structure using successive EPD of NiO-GDC, GDC and LSCF-GDC layers onto a graphite rod, followed by joint sintering at a temperature of 1500 °C (6 h). During the EPD of the anode and cathode layers, PMMA (polymethyl methacrylate) as a pore former was added to the ethanol-based suspensions, while the EPD of the GDC electrolyte layer was carried out with the addition of a PVB binder. As a dispersant, phosphate ester (PE) was added to the suspensions used. The thickness of the GDC electrolyte layer was 10 μm. A specific power density of ~250 mW cm^−^^2^ at an OCV value of ~0.6 V was achieved at a temperature of 700 °C.

**Figure 17:**
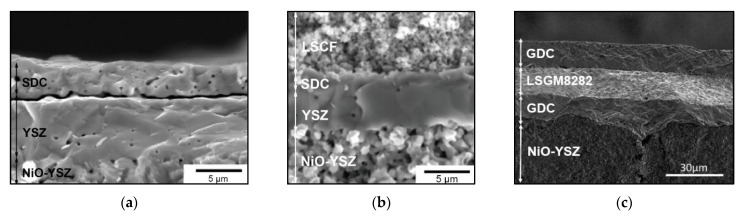
Electrophoretically deposited multilayer electrolytes: two-layer YSZ/SDC electrolyte on an anode substrate NiO-YSZ after co-sintering at 1400 °C, 2 h—(**a**) delamination between the SDC and YSZ layers and (**b**) without delamination at the SDC/YSZ interface due to a decrease in the SDC thickness to 1 μm [137]; (**c**) three-layer GDC/LGSM/GDC electrolyte co-sintered with a NiO-YSZ anode substrate at 1400 °C, 2 h [138].

It should be noted that electrophoretically formed cells with single-layer CeO_2_-based electrolyte membranes possess a reduced OCV value relative to the theoretical Nernst potential, which is due to the properties of cerium dioxide as an MIEC electrolyte with oxygen-ionic and electronic conductivity [8]. The solution to the problems of both internal short circuiting and electrode/electrolyte interaction was demonstrated in the work of Matsuda et al. [137], in which the sequential EPD of YSZ and SDC layers was carried out on a NiO-YSZ substrate followed by joint sintering at a temperature of 1400 °C (2 h). The authors noted the appearance of delamination between the YSZ and SDC layers during the sintering which was eliminated by reducing the thickness of the SDC film to 1 µm (Figure 17a and Figure 17b, respectively).

For the single cell with the two-layer YSZ (4 μm)–SDC (1 μm) electrolyte and an LSFC cathode, a specific power of ~600 mW cm^−2^ and an OCV value of ~1.1 V at a temperature of 700 °C were achieved. The Uchikoshi group [138] fabricated a GDC/LSGM/GDC three-layer electrolyte (where LSGM is La_0.8_Sr_0.2_Ga_0.8_Mg_0.2_O_3−δ_) on a NiO–YSZ supporting anode. Green plates of NiO-YSZ were prepared by slip casting, pre-sintered at 900 °C for 2 h and then coated by polypyrrole via chemical oxidation and polymerization of pyrrole. The electrolyte layers were deposited one after another (the previous layer was kept wet before the deposition of the next layer) and then co-sintered at 1400 °C. Despite that fact that the thickness of each layer was ~10 µm, no cracks and no delamination of the layers were observed (Figure 17c). The OCV of the cell with the three-layer electrolyte and Sm_0.5_Sr_0.5_CoO_3_ (SSC) cathode was 1.112 V at 600 °C, which is close to the theoretical value of 1.135 V and thus reveals the tightness of the electrolyte layer. However, the cell performance was low due to the chemical interaction of the GDC and LGSM layers at a high sintering temperature with the formation of the low conducting SrLaGa_3_O_7_ phase. Another probable reason for the low cell performance was the high polarization resistance of the supporting anode due to its low porosity. The substrate properties were regulated using rice starch as a pore former in the next study by the group [139]. The use of the Sr-deficient LSGM was proposed to decrease interactions with GDC [140].

Thus, a significant problem in the creation of both single-layer and double-layer electrolyte membranes using doped CeO_2_ is its low sinterability, which requires sintering at a high temperature to obtain a gas-tight film. However, an increase in the sintering temperature leads to undesirable interaction of the layers with the formation of non-conductive phases, as well as cracking or delamination of the film structure due to different shrinkage characteristics of materials during the sintering [141].

A decrease in the sintering temperature of films of doped cerium dioxide obtained by the EPD method can be achieved by introducing sintering additives. For example, Hu et al. [142] used a sintering additive of 2 mol% FeO_1.5_ when sintering a GDC layer deposited on a dense supporting electrolyte YSZ, which made it possible to obtain a dense film 5–8 µm thick at a sintering temperature of 1300 °C for 4 h. In a recent study, Hu et al. [109] considered possible mechanisms of charge transfer of particles and ions in a GDC suspension and features in increasing the deposition weight during the EPD. The authors carried out a comparative study of the deposition of GDC particles in an ethanol-based suspension on a PPy-coated YSZ substrate and on a graphite substrate. In particular, it was shown that the rate of deposition of the GDC layer on the PPy surface was lower than that on graphite at the same voltage, which was attributed by the authors to the lower conductivity of PPy. A mechanism was proposed for the appearance of a region saturated with H^+^ ions near the cathode. When the deposit grew, the concentration of protons in the suspension in this region decreased, which was confirmed by direct measurements of the local pH value near the cathode. This effect, as suggested by the authors of the work, is associated with the processes of desorption of protons adsorbed on the particles during the formation of the precipitate, the reduction of protons at the cathode with the release of molecular hydrogen and the decomposition of trace amounts of water in the suspension. The significant role of the diffusion redistribution of protons in the porous deposit and the migration of free H^+^ ions in the suspension are also discussed. It should be noted that the authors added a significant amount of molecular iodine to the suspension; however, the EPD mechanism that they considered was with the participation of protons only. It can be assumed that the migration of I^−^ ions and their interaction with the ionic atmosphere around the particles can affect both the charge transfer and the process of particle deposition on the substrate [143]. In a recent work, Hu et al. [144] conducted a study of methods of conducting EPD using alternating voltage with different amplitudes and durations of positive and negative intervals. It was shown that the use of an asymmetric alternating voltage (–100 V/80 V) during the EPD resulted in the density of the obtained GDC coatings increasing due to the denser packing of particles in the deposit and elimination of the evolution of molecular hydrogen. Based on the analysis of the morphology of the coatings sintered at 1350 °C (4 h), the best results were obtained at a voltage frequency of 500 Hz. To improve the sintering of the obtained coatings, a sintering additive of 2 mol% FeO_1.5_ was also introduced, as noted in the previous work of the group [142].

The challenges arising from the EPD formation of single- and two-layer electrolyte coatings on the surface of non-conductive NiO-SDC anode substrates, as well as the issues in creating various conductive sublayers on the anode surface were considered in a recent study by Kalinina et al. [145]. The formation of a dense sintered SDC layer without sintering additives was shown to be achieved at a temperature of 1550 °C for 5 h. It was demonstrated that the method of creating the conductivity of the anode substrate surface by applying finely dispersed platinum has significant technological capabilities, which allows further multiple EPD of layers with their pre-sintering. In this case, when using the known method of applying a conductive layer of polypyrrole, it is necessary to repeat its synthesis on the surface of the pre-sintered electrolyte layer before applying the next layer. Repetition of PPy synthesis can be avoided using sequential EPD of the layers with following joint co-sintering [137,138].

**Figure 18:**
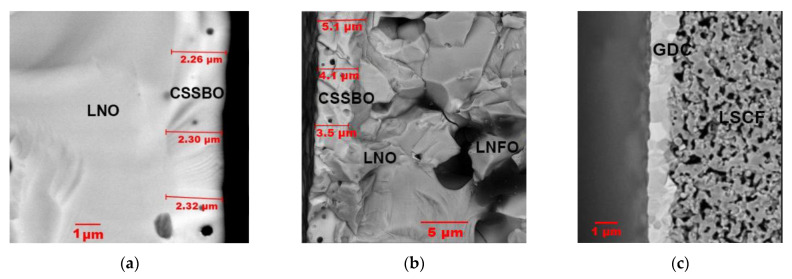
Electrolyte layers, electrophoretically deposited on cathode substrates: (**a**) Ce_0.8_(Sm_0.75_Sr_0.20_Ba_0.05_)_0.2_O_2−δ_ (CSSBO) electrolyte deposited on the single-layer dense La_2_NiO_4+δ_ (LNO) [107] and (**b**) on the two-layer porous LNO/LNF (LaNi_0.6_Fe_0.4_O_3−δ_) cathode substrates and sintered at 1400 °C [108]; (**c**) Ce_0.9_Gd_0.1_O_1.95_ (GDC) electrolyte deposited the two-layer porous (La_0.6_Sr_0.4_)_0.95_Co_0.2_Fe_0.8_O_3−δ_ (LSCF) cathode substrate with supporting and functional layer (CFL) and sintered at 1000 °C (reconstructed from Ref. [149]).

Examples of obtaining dense thin-film coatings of a multi-doped solid electrolyte of Ce_0.8_(Sm_0.75_Sr_0.20_Ba_0.05_)_0.2_O_2−δ_ (CSSBO) on supporting cathode substrates at a relatively low temperature (1400 °C) are presented in the studies performed by Kalinina et al. [107,108]. The coatings were obtained from self-stabilizing suspensions in isopropanol and isopropanol/acetylacetone (50/50 vol%) media prepared on the base of nanopowders with an average particle size of 15 nm, obtained by a laser evaporation/condensation method [146]. To carry out the EPD, the CSSBO suspensions with a concentration of 10 g/L were used with the addition of a polymer binder BMC-5 (copolymer of butyl methacrylate with 5 mol% methacrylic acid) in order to exclude cracking of the coatings during drying. In the initial study [107], a 2.3 μm thick CSSBO coating was obtained in one EPD cycle on a dense model substrate La_2_NiO_4+__δ_ (LNO) (Figure 18a), while in the following work [108], the cyclic EPD of the CSSBO film was carried out on a porous LNO cathode substrate, and a film thickness of 5 μm was achieved during sintering at a temperature of 1400 °C for 4 h (Figure 18b). In [147], the same group modified the cathode substrates in order to enhance their functional properties. The influence of the specific surface area of the starting electrode powders, the introduction of graphite as a pore-forming agent and the sintering temperature of the substrate layers on their porosity, gas tightness, and electrical conductivity was studied. The cyclic EPD of a multi-doped Ce_0.8_(Sm_0.8_Sr_0.2_)_0.2_O_2−δ_ (CSSO) electrolyte with a thickness of 5 µm was performed on an optimized two-layer LNO/LNF cathode substrate. It was shown that the cathode substrate of the optimized design preserved its porosity of approximately 38% after all cycles of the electrolyte sintering, making it favorable for SOFC applications.

Yamamoto et al. [148] developed a method for obtaining a dense thin-film GDC electrolyte electrophoretically deposited on a porous cathode substrate (La_0.6_Sr_0.4_)_0.95_Co_0.2_Fe_0.8_O_3−δ_ (LSCF) by anomalously low-temperature sintering (at 1000 °C), based on the synergetic effect of using highly sinterable GDC nanocubes and the promoted shrinkage of LSFC cathode substrates that contain a sintering additive. GDC nanocubes with reactive {100} facets with an average size of 10 nm were obtained by the organic ligand-assisted hydrothermal method [149]. Enhanced densification of the LSFC substrates at a decreased temperature was adjusted using A-deficient material, as well as using fine LSFC powder, prepared by carbonate co-precipitation, and by the introduction of a sintering additive of 1.5 mol% CuO. The authors compared the sinterability of coatings deposited using nanocubic GDC and conventional GDC nanoparticles (20 nm); in the case of the latter, a dense film structure was not obtained. Experiments performed on the LSFC substrates, prepared from the LSFC commercial powder and pre-sintered at 1100 °C, 2 h showed that the EPD of nanocubic GDC followed by sintering allowed a dense coating to form, while a GDC electrolyte layer obtained by spin-coating and sintered at the same temperature was porous. A gas-tight electrolyte layer of superior quality from GDC nanocubes was obtained using EPD followed by co-sintering at 1000 °C with the LSFC substrate prepared from the fine precipitated powder, with the addition of CuO, spin-coated with a functional cathode layer and pre-sintered at 500 °C, thus exhibiting high shrinkage. The integrated GDC electrolyte/LSCF cathode supports fabricated in the study at anomalously low sintering temperature are suitable for low-temperature solid oxide fuel cells.

As a conclusion, it should be noted that EPD on the cathode substrate has some advantages with regards to the deposition on the anode substrates. First of all, cathode substrates possess high levels of surface conductivity and there is no necessity for the creation of additional conductive layers. In addition, compared to oxide cathode materials, conductive Ni-cermet anode substrates exhibit significant shrinkage during oxidative sintering which may cause delamination of the deposited thin films. All these disadvantages of deposition on cermet anodes stimulate interest in deposition on cathode substrates and oxide anodes, which have been intensively studied recently [150].

## 5. Environmental and Economic Aspects of the Application of Electrodeposition in SOFC Technology

The creation of hydrogen-fueled SOFC-based power plants that could be stable and suitable for long-term operation in the intermediate and low-temperature region will further the elimination of CO_2_ emissions and carbon pollution of the atmosphere, and will also create an alternative to lithium-ion batteries—the massive use of which causes huge problems with their recycling and disposal [151,152,153]. A comparative analysis of the characteristics and technological principles of electrochemical devices for the conversion and storage of energy, including lithium-ion batteries, supercapacitors and fuel cells, was carried out in a recent review work [154]. Various aspects of the transition to the renewable energy technologies with zero CO_2_ emissions were analyzed by Peksen [155] who highlighted the current challenges and opportunities for the transition to new vehicles based on environmentally friendly hydrogen power plants. Methodological aspects of the life-cycle sustainability of SOFCs were considered by Mehmeti et al. [156]. The authors compared SOFCs with other technologies competing for electricity production and substantiated their advantages from the environmental perspective. They also revealed that, due to their superior energy efficiency, SOFCs are economically competitive even when their production costs are higher than those of traditional systems.

The current trend towards lowering the operating temperatures of SOFCs aims to reduce the degradation of elements and replace the noble metals used in high-temperature devices with cheaper materials. However, a decrease in operating temperatures leads to a decrease in the thermoactivated electrical properties of the cell components—anode, cathode and electrolyte [157]. In addition, the low temperature cells have also experienced significant coking, further reducing productivity. New technologies are needed to reduce the cost of SOFCs without sacrificing productivity. EPD that does not require expensive equipment and allows precise control over the coating thickness offers advantages in the deposition of thin-film electrolytes to lower ohmic losses in the cells with reduced operating temperature [1,18]. This technique is based on the application of an electric field and is suitable for deposition on the substrates of complex shapes, as well as for covering the selected areas of the cells to create barrier and protective coatings. It should be noted that the development of new technologies for the electrodeposition of ceramic coatings, particularly those based on cerium dioxide, can create an alternative to the existing technologies for the electrolytic deposition of protective chromium coatings associated with the use of toxic electrolytes containing Cr (VI) [158].

A significant reduction in costs and a decrease in the turnover of hazardous reagents is possible when replacing the process of infiltration of active electrodes with a more efficient and economical one-step ELD process [93]. The energy consumption for the ELD with currents of 20 mA and voltages < 1 V is minimal (~20 mW during deposition), while impregnation has to be repeated many times to achieve percolation (usually 10–20 steps), with a heat treatment at approximately 500–800 °C after each impregnation. A typical furnace has a power consumption that is several kWs greater than the required 20 mW for the ELD process of the electrode activation. Thus, widespread implementation of the electrodeposition methods will shorten the technological cycle of SOFC production and reduce the negative impact on the environment throughout the entire life cycle of electrochemical power plants—from development, manufacture and operation through to their disposal. The cost of low-temperature SOFCs fueled with syngas and methane can be reduced by using cheap and commercially available nickel foam electroplated with Cu instead of Au mesh current collectors [101]. Therefore, the usage of electrodeposition can provide economic benefits for the large-scale commercialization of such electrochemical devices.

## 6. Conclusions

Modern research in the field of solid oxide fuel cells is focused on solving the following problems: increasing the specific power, reducing the operating temperature, optimizing the technology and the successful commercialization and industrial implementation of SOFCs. Electrodeposition methods have several inherent, significant advantages, including the low cost of the applied technological equipment, the absence of vacuum processes, the possibility of mass industrial production and the flexibility of regulating the parameters of the deposited layers. Another factor that interests researchers in electrodeposition technology (both ELD and EPD) is the search for ways to improve the functional characteristics of the main SOFC elements—the solid-state electrolyte and the electrodes. Electrochemical synthesis techniques such as anodic oxidation, cathodic reduction and alternating current synthesis are seen as being a cost-effective alternative to sputtering methods for obtaining nanostructured SOFC cathodes due to the low sintering temperature of the ELD films. Electrodeposition can also replace known impregnation technology, widely used for increasing electrode electrochemical activity by expanding the TPB and improving contacts on the electrode/electrolyte interface. In contrast to impregnation, which usually requires 10 or more technological steps, the necessary amount of material can be introduced into the porous backbone by ELD in one deposition cycle. Chemically assisted electrodeposition (CAED) is a notable and relatively new technique for fabricating nanostructured SOFC cathodes, which includes the deposition of mixed-metal hydroxides on a carbon nanotube template, followed by low-temperature sintering. Lowering the temperature not only results in electrodes with extended TPB, but also reduces interactions between the electrolyte on the interface with the electrodes as well as with the composite electrodes. The performance of single cells with nanofibrous electrodes obtained by CAED is more than double that of cells with conventional electrodes.

ELD can be applied to the deposition of the protective thin-film coatings on SOFC electrodes and interconnects to increase their durability and to create composite electrodes based on Pt and Ag, with a core-shell structure for low-temperature applications. Metallizing of the porous electrolyte scaffolds and surface modification of Ni-based foams using ELD are known methods to increase SOFC anode performance and enhance coking resistance. EPD is suitable for the deposition of thick-film electrodes with regulated porosity and an oriented structure. The advantages of EPD also include the possibility of obtaining films from multicomponent materials and composites, including those with one-dimensional materials. Characteristics of SOFCs with electrodes modified using electrodeposition methods, compared to those without modification or prepared using conventional infiltration technique, are presented in Table 1.

Along with the advantages, the following key problems that impede the implementation of electrodeposition methods in SOFC technology, and whose ultimate resolution necessitates further study, are highlighted in the review:

- Difficulties associated with ELD of multicomponent coatings;
- The complexity of identifying the structure and phase content of the resulting coatings;
- High sensitivity to the conditions and modes of electrodeposition and non-reproducibility of results in some cases;
- The formation of loose deposits and the formation of cracks and pores in the CeO_2_-based coatings obtained using cathodic ELD. The problem of cracking of electrodeposited coatings can be partially solved by co-deposition with a binder (for example, PDDA) or by the electrochemical synthesis of films of doped cerium dioxide;
- The challenges in forming the multilayer and composite coatings;
- The need to create the surface electrical conductivity of the non-conductive substrate for the implementation of the electrodeposition process;
- The necessity of loweing the sintering temperature of the EPD coatings to preserve the porosity of the carrying substrates (SOFC electrodes).

In summary, the most likely areas for utilizing the ELD method are expected to be in the field of creating protective coatings and highly active electrodes for intermediate and low-temperature SOFCs, while the creation of gas-tight solid electrolyte layers and thick-film electrodes, including those based on multicomponent materials and composites with a thickness of 1 μm or more, can be successfully implemented using the EPD method. Further research is required in this area as well as a search for opportunities for integration of various thin-film technologies, including electrodeposition, for low-cost and time-effective production of SOFC structures.

## Figures and Tables

**Table 1:** Comparative characteristics of some single SOFCs/symmetric cells with electrodes modified using electrodeposition, and those with electrodes fabricated using conventional methods (screen printing, sputtering, infiltration). Bold style is used to mark the modified electrode.

Cell Configuration	Power Density, W/cm^2^ (T, °C)	ASR, Ω·cm^2^ (T, °C)	Long-Term Study	Ref.
NiO-YSZ/NiO-ScCeSZ/ScCeSZ/**LCO ^5^-ScCeSZ** (nanofibrous LCO-GDC prepared by CAED) NiO-YSZ/NiO-ScCeSZ/ScCeSZ/LCO-ScCeSZ (screen-printed LCO-ScCeSZ)	0.591 (700) 0.781 (750) 0.956 (800) 0.360 (800)	1.25 (700) 0.93 (750) 0.71 (800) 1.17 (800)	Increase in the cell voltage during the first 20 h from 0.61 to 0.65 V at 1 A/cm^2^ (750 °C). 200 h cell testing, no degradation.	[52]
NiO-YSZ ^1^/NiO-ScCeSZ ^2^/ScCeSZ/**LNO ^3^-GDC ^4^** (LNO-GDC modification by CAED) NiO-YSZ/NiO-ScCeSZ/ScCeSZ/LNO-GDC (LNO-GDC prepared by sintering, without modification)	0.477 (650) 0.717 (700) 0.974 (750) 0.528 (750)	1.632 (650) 0.985 (700) 0.625 (750) 2.557 (650) 1.515 (700) 0.884 (750)	Sharp increase in the cell voltage during the first 24 h. 100 h cell testing at 1 A/cm^2^ (750 °C), no degradation.	[53]
NiO-YSZ/NiO-ScCeSZ/ScCeSZ/**LNF ^6^-GDC** (nanofibrous LNF-GDC prepared using hybrid ELD (electroplating + CAED) NiO-YSZ/NiO-ScCeSZ/ScCeSZ/LNF-GDC NiO-YSZ/NiO-ScCeSZ/ScCeSZ/LSCF ^7^-GDC (screen-printed LNF-GDC and LSCF-GDC)	0.61 (700) 0.98 (750) 1.32 (800) 0.476 (750) 0.874 (750)	0.415 (700) 0.284 (750) 0.223 (800) 0.898 (750) 0.379 (750)	At 0.64 A/cm^2^ (750 °C), Cr-poisoning conditions: 300 h, no degradation. Drop in the cell voltage: 100 h, 6% (LNF-GDC) 100 h, 9.6% (LSCF-GDC)	[54]
**Pt-SDC ^8^**/YSZ/**Pt-SDC** (Pt electrode CELD-covered with SDC) Pt/YSZ/Pt (conventional sputter-deposited Pt electrode)		44 (450) 224 (400) 4810 (450) 38300 (400)	100 h testing at 600 °C, no degradation. 40 h testing, 146% degradation.	[66]
**Ag-PCO ^9^**/YSZ/**Ag-PCO** (Ag electrode CELD-covered with PCO) Ag/YSZ/Ag (conventional sputter-deposited Ag electrode)	0.067 (450) 0.048 (450)	3.7 (450) 121 (450)	100 h testing at 550 °C, no degradation. 70 h testing at 450 °C, initial degradation before stabilization (20 times for 10 min)	[71]
NiO-GDC/GDC/**LN ^10^** Oriented LN deposited by EPD in magnetic field NiO-GDC/GDC/LN Chaotically oriented LN	0.011 (500) 0.008 (500)	12 (500) 25 (500)		[75]
Pd-CeO_2_-YSZ/YSZ/**LSM ^11^**-YSZ LSM-YSZ obtained by EPD Pd-CeO_2_-YSZ/YSZ/LSM-YSZ LSM infiltrated into YSZ backbone			Ohmic losses 0.65 and 0.55 Ω cm^2^ (before and after shorting). Ohmic losses 0.45 Ω cm^2^ (before and after shorting) (700 °C)	[49]
**Cu-Ni-YSZ**/YSZ/LSM-YSZ Cu electroplating Ni-YSZ anode Ni-YSZ/YSZ/LSM-YSZ without modification	0.32 (700) H_2_ 0.24 (700) CH_4_ 0.38 (700) H_2_ 0.28 (700) CH_4_	1.68 (700) H_2_ 2.18 (700) CH_4_ 1.00 (700) H_2_ 1.96 (700) CH_4_	0.17 W/cm^2^ and 2.75 Ω·cm^2^ after 200 h testing using CH_4_ (700 °C) Stepwise degradation and cracking after 21 h	[88]
**Co-Cu–ceria–YSZ**/YSZ/LSM-YSZ 13 vol% Cu, Co modification by ED (5 vol%) Co-Cu–ceria–YSZ/YSZ/LSM-YSZ 13 vol% Cu, Co impregnated (5 vol%) Cu–ceria–YSZ/YSZ/LSM-YSZ 18 vol% Cu, without modification	0.375 (900) 0.120 (900)	0.80 (900) 0.88 (900)	Before/after 50 h testing at 900 °C: Ohmic losses 0.50/0.72 Ω cm^2^ Ohmic losses 0.80/2.00 Ω cm^2^ Ohmic losses 0.88/2.40 Ω cm^2^	[89]
**Ni-GDC**/YSZ/**Ni-GDC** Ni electrodeposited on GDC backbone Ni-GDC/YSZ/Ni-GDC Ni infiltrated GDC		1.00 (700) wet H_2_ 0.168 (700) wet H_2_		[86,93]
**Ni-Ag-GDC**/YSZ/GDC-Ag-Ni Ni electrodeposited on Ag-GDC backbone Ag-GDC/YSZ/GDC-Ag		1.12 (750) wet H_2_ 3.77 (750) H_2_		[94]
**Cu-Ni-foam**-LSCM ^12^-YSZ-Pt Ni-foam collector Cu-modified by plating Au-LSCM-YSZ-Pt	0.155 (900) Syngas^13^ 0.140 (900)	13.5 (900) syngas 15.0 (900)	48 h testing using syngas as a fuel (900 °C), no degradation	[100]

^1^ YSZ—yttria-stabilized zirconia; ^2^ ScCeSZ—scandia-ceria-stabilized zirconia; ^3^ LNO—LaNiO_3_; ^4^ GDC—gadolinium-doped ceria; ^5^ LCO—LaCoO_3_; ^6^ LNF—LaNi_0.6_Fe_0.4_O_3−δ_; ^7^ LSCF—La_0.6_Sr_0.4_Co_0.2_Fe_0.8_O_3−δ_; ^8^ SDC—Sm_0.16_Ce_0.84_O_1.92_; ^9^ PCO—Pr_0.2_Ce_0.8_O_2−δ_; ^10^ LN—La_2_NiO_4+δ_; ^11^ LSM—La_0.8_Mn_0.2_O_3−δ_; ^12^ LSCM—La_0.75_Sr_0.25_Cr_0.5_Mn_0.5_O_3−δ_; ^13^ syngas—40 vol% of H2, 60 vol% of CO and 500 ppm of H2S.
